# Overview of Tensor-Based Cooperative MIMO Communication Systems—Part 1: Tensor Modeling

**DOI:** 10.3390/e25081181

**Published:** 2023-08-08

**Authors:** Gérard Favier, Danilo Sousa Rocha

**Affiliations:** 1 I3S Laboratory, Côte d’Azur University, 06903 Sophia Antipolis, France; 2 Federal Institute of Education, Science and Technology of Ceará, Campus Sobral, Sobral 62042-030, Brazil; danilo.rocha@ifce.edu.br

**Keywords:** cooperative communication systems, MIMO, relaying systems, tensor codings, tensor models

## Abstract

Due to increasingly strong and varied performance requirements, cooperative wireless communication systems today occupy a prominent place in both academic research and industrial development. The technological and economic challenges for future sixth-generation (6G) wireless systems are considerable, with the objectives of improving coverage, data rate, latency, reliability, mobile connectivity and energy efficiency. Over the past decade, new technologies have emerged, such as massive multiple-input multiple-output (MIMO) relay systems, intelligent reflecting surfaces (IRS), unmanned aerial vehicular (UAV)-assisted communications, dual-polarized (DP) antenna arrays, three dimensional (3D) polarized channel modeling, and millimeter-wave (mmW) communication. The objective of this paper is to provide an overview of tensor-based MIMO cooperative communication systems. Indeed, during the last two decades, tensors have been the subject of many applications in signal processing, especially for digital communications, and more broadly for big data processing. After a brief reminder of basic tensor operations and decompositions, we present the main characteristics allowing to classify cooperative systems, illustrated by means of different architectures. A review of main codings used for cooperative systems is provided before a didactic and comprehensive presentation of two-hop systems, highlighting different tensor models. In a companion paper currently in preparation, we will show how these tensor models can be exploited to develop semi-blind receivers to jointly estimate transmitted information symbols and communication channels.

## 1. Introduction

Since the pioneering work [1,2], cooperative multiple-input multiple-output (MIMO) systems have emerged as promising techniques to improve the coverage, data rate, diversity, and performance of wireless communications. Over the past decade, new technologies have been developed, such as massive MIMO relay systems; intelligent reflecting surfaces (IRS), also known as reconfigurable intelligent surfaces (RIS); unmanned aerial vehicular (UAV)-assisted communications; dual-polarized (DP) antenna arrays; three dimensional (3D) polarized channel modeling; and millimeter-wave (mmW) communication.

IRS- and UAV-assisted systems have recently received great attention for their potential to control the ambient environment, to enhance signal coverage, and to reduce the implementation costs and energy consumption of future wireless systems.

Note that, contrary to relays, IRSs that consist of quasi-passive elements are not equipped with hardware to process signals, and therefore, they are not able to carry out decoding and coding operations. IRS-assisted communication systems generally operate in a supervised way, i.e., using a training sequence, for channel estimation. Such systems, which operate similarly to relay-aided systems, have some advantage in terms of spectral and/or energy efficiency gains. A comparison of these two technologies can be found in [3,4].

UAV-aided MIMO communications offer new perspectives for wireless networks and Internet of Things (IoT) applications [5,6]. In such applications, UAVs, also known as drones, can be viewed as mobile relays between IoT devices or users and a base station (BS). UAVs can also be combined with IRS technology to enhance communication performance. For instance, in an urban environment with multiple IRSs, a UAV can be used to assist IRS data transmission to a BS [7]. Multiple UAVs can be employed as aerial mobile base stations to transmit information to ground users with the help of an IRS, aiming to assist terrestrial communication systems, e.g., to offload hotspot cellular traffic [8].

To improve system capacity and spectral efficiency, cooperative MIMO systems use mmW transmission technology, allowing them to achieve gigabit-per-second data rates [9,10].

During the last two decades, numerous wireless communication systems have been designed using tensor-based approaches, with the aim of taking different diversities (space, time, frequency, code, polarization, etc) into account, and developing semi-blind receivers for jointly estimating the channels and transmitted information symbols. The reader is referred to the following work [11] for a survey of such systems.

The purpose of this paper is to provide an overview of tensor-based cooperative communication systems, including relay systems and IRS- and UAV-assisted systems. This overview, which concerns only the tensor modeling aspect, is by no means exhaustive. A companion paper is being prepared [12] for presenting semi-blind receivers allowing to jointly estimate information symbols and individual channels in the context of different cooperative systems. Tensor model uniqueness of each system and parameter identifiability conditions for each receiver will be analyzed and compared. Some Monte Carlo simulation results will be provided for illustrating the performance of the considered systems and receivers.

The main contributions of this paper can be summarized by the following points:

- A detailed introduction of the tensor operations and decompositions useful for designing tensor-based cooperative communication systems; see Section 2.
- An overview of various MIMO cooperative systems, including relaying systems, IRS- and UAV-assisted communication systems; see Section 3.
- An overview of the main codings used in MIMO cooperative systems, with a particular emphasis on tensor codings proposed during the last decade in the context of point-to-point and cooperative communication systems; see Section 4.
- A presentation in a didactic and unified way of several two-hop systems highlighting different new tensor models. Some of these systems are extensions of existing ones; see Section 5.

**Notation:** Table 1 summarizes the notations used in this paper.

## 2. Tensor Prerequisites

In this section, we first review some basic notions like slice, mode combination and tensor matricization. Then, the most important tensor operations and decompositions used throughout the paper are recalled. For complementary information on tensor tools, the reader is referred to [13,14].

### 2.1. Some Definitions and Notion of Slice

In signal processing applications, an *N*th-order tensor $X\in K^{{\underline{I}}_{N}}$, of size ${\underline{I}}_{N}≜I_{1}\times \cdots \times I_{N}$, is an array of real (K=R) or complex (K=C) numbers denoted $X=\left[x_{{\underline{i}}_{N}}\right]=\left[x_{i_{1},\cdots ,i_{N}}\right]$, where ${\underline{i}}_{N}≜\left\{i_{1},\cdots ,i_{N}\right\}$. Each index $i_{n}\in \langle I_{n}\rangle ≜\left\{1,\cdots ,I_{n}\right\}$, for n∈〈N〉≜{1,⋯,N}, is associated with a mode, also called a way. This explains the other appellation-like multiway array for a tensor. The number of indices defines the order of the tensor, and the number of elements in X is equal to $\prod_{n=1}^{N}I_{n}$, where $I_{n}$ denotes the dimension of the *n*th mode. Note that the special cases N=2 and N=1 correspond to the sets of matrices $X\in K^{I\times J}$ and column vectors $x\in K^{I}$, respectively.

The identity tensor of order *N* and size I×⋯×I is denoted $I_{N,I}=\left[\delta_{i_{1},\cdots ,i_{N}}\right]$, with $i_{n}\in \langle I\rangle$, for n∈〈N〉, or simply $I_{I}$. It is a diagonal hypercubic tensor whose diagonal elements are equal to 1 and other ones to zero, defined using the generalized Kronecker delta as:

$$\delta_{i_{1},\cdots ,i_{N}}=\left\{\begin{array}{c} 1\;\mathrm{if}\;i_{1}=\cdots =i_{N}\\ 0\;\mathrm{otherwise} \end{array}\right..$$

The Frobenius norm of $X\in K^{{\underline{I}}_{N}}$ is the square root of the inner product of the tensor with itself, i.e.,:

(1) $${∥X∥}_{F}=\sqrt{\langle X,X\rangle }={\left(\sum_{i_{1}=1}^{I_{1}}\cdots \sum_{i_{N}=1}^{I_{N}}{\left|x_{i_{1},\cdots ,i_{N}}\right|}^{2}\right)}^{1/2}.$$

A slice is a sub-tensor obtained by fixing one or more indices. If we fix N−1 indices of an *N*th-order tensor $X\in K^{{\underline{I}}_{N}}$, we obtain a vector called a fiber. When fixing index $i_{n}$ of X, with n∈〈N〉, we obtain a (N−1) order tensor denoted $X_{\left(i_{n}\right)}\in K^{I_{1}\times \cdots \times I_{n-1}\times I_{n+1}\times \cdots \times I_{N}}$. Thus, for a third-order tensor $X\in K^{I\times J\times K}$, fixing one index gives three types of matrix slices, called horizontal, lateral, and frontal, when the indices *i*, *j*, and *k* are fixed, respectively, and denoted as follows:

(2) $$X_{i..}\in K^{J\times K},X_{.j.}\in K^{K\times I},X_{..k}\in K^{I\times J}.$$

**Figure 1:**
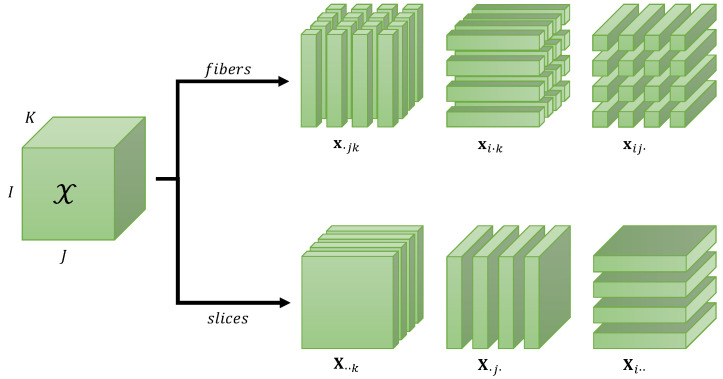
Fibers (column, row, and tube) and matrix slices (frontal, lateral, and horizontal) of a third-order tensor $X\in K^{I\times J\times K}$.

Fibers and matrix slices of a third-order tensor $X\in K^{I\times J\times K}$ are illustrated in Figure 1.

### 2.2. Notion of Mode Combination and Matricization

Mode combination is a very important operation in tensor calculus and can be viewed as a transformation of a tensor of order *N* into a tensor of order $N_{1}<N$. Matricization, also called matrix unfolding, of a tensor is a fundamental operation using mode combination to transform an *N*th-order tensor $X\in K^{{\underline{I}}_{N}}$ into a matrix.

Considering a partitioning of the set of modes S=〈N〉 into two disjointed ordered subsets $S_{1}$ and $S_{2}$, composed of *p* and N−p modes, respectively, with p∈〈N−1〉, a general matrix unfolding formula for an *N*th-order tensor X was given by [15] as:

(3) $$X_{S_{1}\;;\;S_{2}}=\sum_{i_{1}=1}^{I_{1}}\cdots \sum_{i_{N}=1}^{I_{N}}x_{i_{1},\cdots ,i_{N}}\left(\underset{n\in S_{1}}{⊗}e_{i_{n}}^{\left(I_{n}\right)}\right){\left(\underset{n\in S_{2}}{⊗}e_{i_{n}}^{\left(I_{n}\right)}\right)}^{T}\in K^{J_{1}\times J_{2}},$$

where $e_{i_{n}}^{\left(I_{n}\right)}$ is the $i_{n}$-th vector of the canonical basis of $R^{I_{n}}$, and $J_{n_{1}}=\underset{n\in S_{n_{1}}}{\prod I_{n}}$, for $n_{1}=1\;\mathrm{and}\;\;2$. We say that $X_{S_{1}\;;\;S_{2}}$ is a matrix unfolding of X along the modes of $S_{1}$ for the rows and along the modes of $S_{2}$ for the columns, with $S_{1}\cap S_{2}=\emptyset$ and $S_{1}\cup S_{2}=\langle N\rangle$.

For instance, in the case of a third-order tensor $X\in K^{I\times J\times K}$, we have six flat unfoldings and six tall unfoldings. For $S_{1}=1$ and $S_{2}=\left\{2,3\right\}$, we have the following mode-1 flat unfolding $X_{I\times JK}≜X_{1\;;\;\{2,3\}}$, while for $S_{1}=\left\{2,3\right\}$ and $S_{2}=1$, we obtain the following mode-1 tall unfolding $X_{JK\times I}≜X_{\{2,3\}\;;\;1}$.

By convention, the order of the dimensions in a product $\prod_{p=1}^{P}I_{p}≜I_{1}\cdots I_{P}$ associated with a combination of the indices $\left(i_{1},\cdots ,i_{P}\right)$ follows the order of variation of the indices, with $i_{1}$ varying more slowly than $i_{2}$, which in turn varies more slowly than $i_{3}$, etc. For example, in the matrix unfolding $X_{I\times KJ}$, index *k* varies more slowly than *j*, which implies:

(4) $$x_{ijk}={\left[X_{I\times KJ}\right]}_{i,(k-1)J+j}={\left[X_{J\times IK}\right]}_{j,(i-1)K+k}={\left[X_{K\times JI}\right]}_{k,(j-1)I+i}.$$

**Figure 2:**
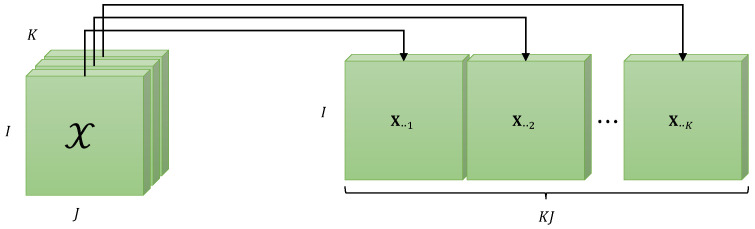
Matrix unfolding $X_{I\times KJ}$ for $X\in K^{I\times J\times K}$.

Figure 2 illustrates the construction of the unfolding $X_{I\times KJ}$ obtained by horizontally stacking the frontal slices $X_{..k}$, for k∈〈K〉.

### 2.3. Recall of Some Tensor Operations

In Table 2, we present two multiplications with tensors, called mode-*p* or Tucker product, and mode-(p,n) product, denoted $\times_{p}$ and $\times_{p}^{n}$, respectively.

The mode-*p* product of a tensor $X\in K^{{\underline{I}}_{P}}$ with a matrix $A\in K^{J\times I_{p}}$, denoted $X\times_{p}A$, corresponds to a summation over the index $i_{p}$ associated with the mode *p* of X and the second index of A.

The mode-(p,n) product of two tensors $X\in K^{{\underline{I}}_{P}}$ and $Y\in K^{{\underline{J}}_{N}}$ corresponds to a contraction operation performed for arbitrary modes (p,n) of X and Y, with $I_{p}=J_{n}=K$. This multiplication gives a tensor Z of order P+N−2 and size $I_{1}\times \cdots \times I_{p-1}\times I_{p+1}\times \cdots \times I_{P}\times J_{1}\times \cdots \times J_{n-1}\times J_{n+1}\times \cdots \times J_{N}$. These two products can be carried out using matrix unfoldings of the tensors involved in the products, as illustrated in Table 3. The resulting tensor is then obtained by means of a reshaping operation based on the definition of the dimensions for the tensor.

**Remark** **1.** *The mode-p and mode-(p,n) products satisfy the following properties.*

- *For two products of $X\in K^{{\underline{I}}_{P}}$ along the mode p, with $A\in K^{J_{p}\times I_{p}}$ and $B\in K^{K_{p}\times J_{p}}$, we have:*
(5) $$Y=X\times_{p}A\times_{p}B=X\times_{p}\left(BA\right)\in K^{I_{1}\times \cdots \times I_{p-1}\times K_{p}\times I_{p+1}\times \cdots \times I_{P}}.$$
  *From this property, we can conclude that the double mode-p product is commutative only if the matrices A and B commute (AB=BA).*
- *The contracted product $\times_{p}^{n}$ is associative; in other words, for any tensors $A\in K^{{\underline{I}}_{P}}$, $B\in K^{{\underline{J}}_{N}}$, and $C\in K^{{\underline{K}}_{Q}}$ such that $I_{p}=J_{n}$ and $J_{m}=K_{q}$, with m≠n, we have:*
(6) $$\left(A\times_{p}^{n}B\right)\times_{m}^{q}C=A\times_{p}^{n}\left(B\times_{m}^{q}C\right)=A\times_{p}^{n}B\times_{m}^{q}C.$$
  *This double contracted product corresponds to a double summation over the indices $i_{p}$ and $j_{n}$ on the one hand, and over the indices $j_{m}$ and $k_{q}$ on the other hand. It provides a tensor of order P+N+Q−4.*
- *Property (6) is valid when (m,n,p,q) bijectively positions the indices $\left(j_{m},j_{n}\right),i_{p}$ and $k_{q}$ in the sets ${\underline{j}}_{N}$, ${\underline{i}}_{P}$ and ${\underline{k}}_{Q}$, respectively. When (m,n,p,q) represent mode numbers, the property (6) is no longer valid because the result of the double-contracted product $\times_{p}^{n}$ and $\times_{m}^{q}$ depends on the order in which these products are made. For instance, for $A\in K^{I_{1}\times J_{1}\times R_{1}}$, $B\in K^{R_{1}\times I_{2}\times J_{2}\times R_{2}}$ and $C\in K^{R_{2}\times I_{3}\times J_{3}}$, the double product can be written in two different ways, giving the same result:*
(7) $$\left(A\times_{3}^{1}B\right)\times_{5}^{1}C=A\times_{3}^{1}\left(B\times_{4}^{1}C\right)\in K^{I_{1}\times J_{1}\times I_{2}\times J_{2}\times I_{3}\times J_{3}}.$$
  *In the left member of this equality, the product $\times_{3}^{1}$ is first computed, followed by the product $\times_{5}^{1}$, while in the right member, the product $\times_{4}^{1}$ is computed before the product $\times_{3}^{1}$. We will say that in the first case (resp. the second case), the modes-(1,p) products are performed from left to right (resp. from right to left). This type of consideration is useful for writing the equation of a tensor train decomposition (TTD). See [16].*

Table 4 presents a few examples of outer products of vectors, matrices and tensors, indicating the space to which the tensors resulting from these products belong, as well as their order.

**Remark** **2.**

- *The outer product of P non-zero vectors $u^{\left(p\right)}\in K^{I_{p}}$, p∈〈P〉 gives a rank-one tensor of order P and size ${\underline{I}}_{P}$ such that:*
(8) $$\overset{P}{\underset{p=1}{\circ }}u^{\left(p\right)}\in K^{{\underline{I}}_{P}}\;\;\;\Leftrightarrow \;\;\;{\left(\overset{P}{\underset{p=1}{\circ }}u^{\left(p\right)}\right)}_{{\underline{i}}_{P}}=\prod_{p=1}^{P}u_{i_{p}}^{\left(p\right)}.$$
  *For instance, the outer product of three non-zero vectors $u\in K^{I},v\in K^{J}$, and $w\in K^{K}$ gives a rank-one, third-order tensor u∘v∘w of size I×J×K such that:*
(9) $${\left(u\circ v\circ w\right)}_{ijk}=u_{i}v_{j}w_{k}\;\;\;,\;\;\;i\in \langle I\rangle ,j\in \langle J\rangle ,k\in \langle K\rangle .$$
  *Reciprocally, a Pth-order tensor A is a rank-one tensor if it can be written as the outer product of P vectors $u^{\left(p\right)}$, with p∈〈P〉. Hitchcock [17] showed that any tensor can be written as a sum of rank-one tensors, and the rank R of the tensor is the smallest number of rank-one tensors needed to write it as a linear combination, i.e.,: $A=\sum_{r=1}^{R}a_{r}^{\left(1\right)}\circ \cdots \circ a_{r}^{\left(P\right)}$, where $a_{r}^{\left(p\right)}$ is the rth column of the pth matrix factor $A^{\left(p\right)}$, p∈〈P〉. As shown in Section 2.4, when R is minimum, such a decomposition is called a canonical polyadic decomposition (CPD) or parallel factor (PARAFAC) analysis [18].*
- *Note that the outer product of $A\in K^{{\underline{I}}_{P}}$ with $B\in K^{{\underline{J}}_{N}}$ gives a rectangular tensor of order P+N belonging to the space $K^{{\underline{I}}_{P}\times {\underline{J}}_{N}}$.*

Table 5 gives expressions for scalar elements of each tensor resulting from the outer products in Table 4.

### 2.4. Recall of Basic Tensor Decompositions

Tensor decompositions, also called tensor models, are used to represent data tensors by means of matrix factors and lower-order tensors, called core tensors. Many different tensor models exist. Several of them have been developed through the design of new wireless communication systems, as illustrated in this paper. In this section, we present the Tucker and PARAFAC decompositions.

In Table 6, we give various forms of representation for these two decompositions in the case of an *N*th-order tensor $X\in K^{{\underline{I}}_{N}}$: scalar writing, writings with mode-*n* products and outer products, and general Formula (3) for matrix unfolding.

**Remark** **3.** *The Tucker decomposition can be viewed as a generalization of the PARAFAC decomposition that takes into account all the interactions between the columns of the matrix factors $A^{\left(n\right)}\in K^{I_{n}\times R_{n}}$ via the introduction of a core tensor $G\in K^{{\underline{R}}_{N}}$. Contrary to PARAFAC, which has the essential uniqueness property under mild conditions, the Tucker decomposition is not unique in general.*

In Table 7, the case of a third-order tensor $X\in K^{I\times J\times K}$ is considered.

A Tucker-$\left(N_{1},N\right)$ model for an *N*th-order tensor $X\in K^{{\underline{I}}_{N}}$ with $N\geq N_{1}$ corresponds to the case where $N-N_{1}$ factor matrices are equal to identity matrices [19].

For example, if we assume that $A^{\left(n\right)}=I_{I_{n}}$, which implies $R_{n}=I_{n}$, for $n=N_{1}+1,\cdots ,N$, and hence $G\in K^{R_{1}\times \cdots \times R_{N_{1}}\times I_{N_{1}+1}\times \cdots \times I_{N}}$, then the equations of the Tucker model in Table 6 become:

(10) $$\begin{array}{cc} x_{i_{1},\cdots ,i_{N}}= & \;\sum_{r_{1}=1}^{R_{1}}\cdots \sum_{r_{N_{1}}=1}^{R_{N_{1}}}g_{r_{1},\cdots ,r_{N_{1}},i_{N_{1}+1},\cdots ,i_{N}}\prod_{n=1}^{N_{1}}a_{i_{n},r_{n}}^{\left(n\right)}, \end{array}$$

(11) $$\begin{array}{cc} X= & \;G\times_{1}A^{\left(1\right)}\times_{2}\cdots \times_{N_{1}}A^{\left(N_{1}\right)}\times_{N_{1}+1}I_{I_{N_{1}+1}}\cdots \times_{N}I_{I_{N}} \end{array}$$

(12) $$\begin{array}{cc} = & \;G\;\overset{N_{1}}{\underset{n=1}{\times }}A^{\left(n\right)}. \end{array}$$

For a third-order tensor $X\in K^{I\times J\times K}$, two special cases are given by the Tucker-(2,3) and Tucker-(1,3) models, often called Tucker2 and Tucker1, respectively. These models are obtained by fixing one or two of the matrix factors equal to identity matrices.

Table 8 summarizes the equations of the Tucker-(2,3) and Tucker-(1,3) models in the case where $C=I_{K}$ for Tucker-(2,3) and $\left(B=I_{J},C=I_{K}\right)$ for Tucker-(1,3).

## 3. Overview of Cooperative Communication Systems

In Section 3.1, we first present how tensor-based cooperative systems can be classified. Then, in Section 3.2, different architectures of cooperative systems will be described before providing an overview of several systems in Section 3.3.

### 3.1. How to Classify Tensor-Based Cooperative Systems

**Figure 3:**
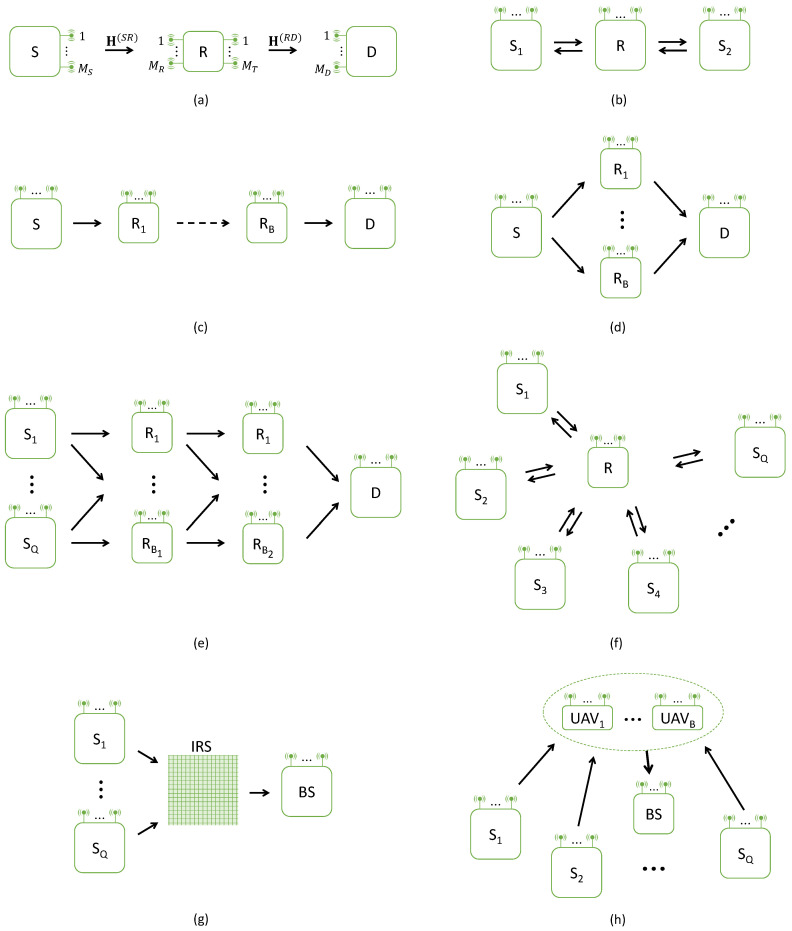
Cooperative systems: (**a**) one-way two-hop; (**b**) two-way two-hop; (**c**) one-way multi-hop; (**d**) one-way two-hop multi-relay in parallel; (**e**) one-way three-hop multi-user multi-relay in parallel; (**f**) two-way multi-user single-relay; (**g**) one-way two-hop multi-user IRS-assisted; (**h**) one-way two-hop multi-user multi-UAV-assisted.

Tensor-based cooperative wireless communication systems can be classified according the following characteristics:

- the network architecture, which depends on the numbers of users (*Q*), hops (*H*) and relays (*B*), as illustrated in Figure 3;
- the modulation technology used in terms of channel access and multiplexing, like CDMA (code division multiple access), TDMA (time-division multiple access), FDMA (frequency-division multiplexing access), OFDM (orthogonal frequency-division multiplexing), or hybrid technique combining OFDM with CDMA, i.e., OFDM-CDMA, also denoted OFCDM;
- the type of coding (matrix/tensor) used at the source and relay nodes, which makes it possible to take into account several diversities, like space-time (ST) and space-time-frequency (STF) codings, to obtain space, time and frequency diversities, i.e., redundancies of transmitted symbols in each of these domains; see Section 4 for a presentation of different codings;
- the type of communication in the sense of two-way versus one-way communication, i.e., with or without feedback from the receiver to the sender;
- the type of transmission in the sense of full-duplex (FD) versus half-duplex (HD) transmission, i.e., using a bi-directional communication channel that can carry information in both directions simultaneously or not, respectively; FD increases system throughput;
- the relaying protocol: the two most common protocols are decode-and-forward (DF) and amplify-and-forward (AF) ones, depending upon the relays decode or not the received signals; with the DF protocol, the signals received at the relays are decoded and then re-encoded before being forwarded to the destination, whereas with the AF protocol, the received signals are simply amplified and retransmitted without decoding;
- the use of a pilot (also called training) sequence at the receiver for channel estimation, which corresponds to a pilot-assisted transmission resulting in a supervised system, in contrast with an unsupervised or semi-blind one when only few pilot symbols are used;
- the type of channel fading in frequency domain: frequency-flat fading versus frequency-selective fading, based on whether or not all frequency components of the transmitted signals are attenuated by the same fading. In the last case, the channel coefficients depend on the frequency. Very often, a block fading is considered, i.e., the channel coefficients are assumed to be constant during a transmission block; they can be time varying when the transmitter and receiver are moving with respect to each other; channel characteristics also concern the presence or non-presence of multipath propagation, and of directional angles (direction of departure (DoD) and of arrival (DoA) angles); other channel properties can be exploited, such as sparsity, low-rank, or reciprocity between forward and backward paths, i.e., between two communication nodes; this property, commonly used in time-division duplexing (TDD) communication networks, allows alleviating overhead requirements for channel state information (CSI) feedback; see [20] for a study of channel reciprocity in IRS-assisted wireless networks;
- the possibility or not of exploiting a direct link between the source and the destination nodes, also called a direct line-of-sight (LOS) path, which is often assumed to be unavailable due to the presence of large obstacles or long distances;
- the tensor models for signals received at the relay and destination, which conditions the type of receiver; the order of the tensors mainly depends on the diversities taken into account via the coding; in Table 9 (presented in Section 3.3), the tensor model associated with each cooperative system is mentioned; a list of main tensor models used in the context of cooperative communications is given at the end of this section;
- the type of receiver: SVD-based closed-form, like the Khatri-Rao factorization (KRF) and Kronecker factorization (KronF) methods, versus iteratives like alternating least-squares (ALS) or Levevenbergh-Marquardt (LM) algorithms.
- the use of intelligent reflecting surface (IRS), i.e., IRS-assisted communication systems, which can be viewed as relay systems employing 2D surfaces composed of a large number of passive reflecting elements for enhancing the coverage of wireless communications;
- the use of unmanned aerial vehicular (UAV), leading to UAV-aided communication systems.

### 3.2. Different Architectures of Cooperative Systems

In Figure 3a–h, we present several architectures of relay-, IRS- and UAV-assisted communication networks. Figure 3a represents a conventional MIMO one-way two-hop relay system, composed of three nodes associated with a source (S) transmitting its information to a destination (D), via a relay (R), as in [21,22,23,24,25]. The source and destination nodes are equipped with $M_{S}$ and $M_{D}$ antennas, respectively, whereas the relay uses $M_{R}$ antennas for reception and $M_{T}$ for transmission.

The channel of the link between the source and relay nodes is denoted $H^{\left(SR\right)}\in C^{M_{R}\times M_{S}}$. Similarly, for the link relay—destination, the channel is denoted $H^{\left(RD\right)}\in C^{M_{D}\times M_{T}}$; see Section 5.1, Section 5.2 and Section 5.3 for the presentation of three examples of two-hop relay systems with a single user.

A two-way two-hop relay system is illustrated in Figure 3b, which corresponds to two users/sources exchanging information via a relay, as in [26,27]. In [26], one of the pioneering works on tensor-based approaches for cooperative communications, the authors propose a two-way relaying system where each user sends a training sequence for partial channel estimation. The relay combines the received signals and retransmits this combination after amplification using an AF tensor that satisfies a CPD decomposition. The tensors of signals received by each user satisfy a CPD model exploited to estimate the channels by means of the KRF algorithm combined with iterative refinements.

Different architectures of cooperative systems result from various multi-relay and multi-user configurations, as briefly described hereafter.

Use of multi-relay is shown in Figure 3c–e with relays in cascade or/and in parallel, respectively. In the first case, we have a multi-hop relaying system [28,29,30,31], while the second case corresponds also to a two-hop system with several relays in parallel [32,33,34]. In [35], a three-hop relaying system is considered with two relay groups (GR1 and GR2), as illustrated in Figure 3e. Individual channels are estimated using a training sequence sent by the source (S) to the destination (D), with a transmission protocol composed of three phases: (1) S→GR1andGR2; (2) GR1→GR2andD; (3) GR2→D, which seems a bit complicated from a synchronization point of view. See Section 5.5 for an example of a system with parallel multi-relay.

In the multi-user case, we distinguish the configurations with a single relay or a single IRS and multiple UAVs, as illustrated in Figure 3f–h, respectively. A multi-user two-way massive MIMO system with a single relay node, as represented in Figure 3f, is proposed by [36]. All nodes operate in half-duplex mode, with the purpose for each user to estimate the channel matrices and the information signals sent by other users. See Section 5.4 for an example of a multi-user system. Note that the tensor-based approach has recently been considered for the design of cooperative mmW MIMO systems, as in [37,38,39,40].

Until now, little work exists regarding the use of tensorial approaches for the design of IRS- and UAV-assisted systems, as briefly summarized below.

A tensor-based approach for a MIMO communication system composed of a base station (BS) transmitting information to a user terminal (UT) via an IRS is proposed in [41,42]. The multi-user case is considered in [38,43], as illustrated by means of Figure 3g; see Section 5.6 for the presentation of a new tensor-based IRS-assisted system.

In [44], a UAV-assisted IoT communication system is proposed using a simplified KRST coding and a training sequence superimposed to encoded information signals for each user. That leads to a combined nested CPD model for the two components of the tensor of signals received at the BS associated with the training sequence and the encoded information signals, respectively. This model is exploited for joint channel estimation and symbol detection.

### 3.3. Overview of Cooperative Systems

In Table 9, we provide an overview of several cooperative systems highlighting their characteristics in terms of modulation (OFDM), technology (mmWave, IRS, UAV), communication (one-way vs two-way), coding, and tensor models. We also mention the numbers of users (Q), hops (*H*) and relays (*B*). This triplet (Q,H,B) is directly linked with the structure of the cooperative system, as illustrated in Figure 3.

**Table 9:** Overview of cooperative systems.

Ref	OFDM	mmW	IRS	One/Two Way	UAV	*Q* Users	*H* Hops	*B* Relays	Coding Training	Tensor Models
[21]				one-way		1	2	1	Simplified KRST	CPD-PARATUCK
[22,23]				one-way		1	2	1	Simplified KRST	Nested CPD
[24]				one-way		1	2	1	TST	NTD
[25]				one-way		1	2	1	MKRST MKronST	CPD
[26]				two-way		2	2	1	Training	CPD
[27]				two-way		1	2	1	TST	Block Tucker-2
[28]				one-way		1	≥2	≥2	Simplified KRST	Gen. Nested CPD
[29]				one-way		1	≥2	≥2	TST	HONTD
[30]				one-way		1	≥2	≥2	Simplified KRST	PARATUCK
[31]				one-way		≥2	3	≥2	KRST	Nested CPD
[32]				one-way		1	2	≥2	Matrices+Training	CPD
[33]				one-way		1	3	2	TST-CPD	NTD
[34]				one-way		1	2	≥2	TST	Coupled NTD
[35]				one-way		1	3	≥2	Matrices+training	CPD + structured Tucker
[36]				two-way		≥2	2	1	TST	Block Tucker2-CPD
[37]	OFDM	X		one-way		1	2	1	Training	Structured CPD
[38]	OFDM	X	X	one-way		≥2	2		Matrices+training	CPD
[39]		X		one-way		1	2	1	Matrices+training	CPD
[40]		X		one-way		1	1		Simplified KRST	Nested CPD
[41,43]			X	one-way		≥2	2	1	Training	CPD
[42]			X	one-way		1	2	1	Training	CPD
[44]				one-way	X	≥2	2	≥2	Simplified KRST + Training	Nested CPD
[45]				two-way		≥2	2	1	TST	Tucker-2
[46]				one-way		1	3	2	TST	NTD
[47]	OFDM			one-way		1	2	1	TST + Simplified TSTF	Coupled NTD
[48]	OFDM			one-way		2	2	1	TST	TTD
[49]	OFDM			one-way		1	2	1	KRSTF	Nested CPD

Some comments are made below on the cooperative systems considered in Table 9.

- Most relay systems use the AF protocol. However, some use the DF protocol. In [25], closed-form semi-blind receivers are proposed to jointly estimate individual channels and symbol matrices, using multiple Khatri-Rao product-based space-time (MKRST) and multiple Kronecker product-based space-time (MKronST) codings at the source and relay nodes. AF and DF protocols are compared with the estimate-forward (EF) protocol for which the estimated symbol matrices are directly re-encoded without a decoding step. DF and EF protocols provide significant symbol error rate (SER) performance improvements at the cost of additional computational complexity at the relay.
- Various tensor models were developed for representing the signals received at the relay and destination nodes:
  – CPD [25,26,32,38,39,41,42,43];
  – Structured CPD (SCPD) [37];
  – Nested CPD (NCPD) [22,23,31,40,44,49];
  – Generalized nested CPD (GNCPD) [28];
  – Tucker decomposition (TD) [45];
  – Block TD [27,36];
  – PARATUCK [30];
  – CPD-structured TD [35];
  – CPD-PARATUCK [21];
  – Nested TD (NTD) [24,33,46];
  – High-order NTD (HONTD) [29];
  – Coupled NTD (CNTD) [34,47];
  – Tensor train decomposition (TTD) [48];
- In the case of MIMO-OFDM relaying systems, different assumptions are made on the channels. In [47,49], the channels are assumed to be constant and flat Rayleigh fading, i.e., matrices, whereas in [37,38], the channels are fourth-order tensors with two space (antennas) dimensions, one frequency dimension (sub-carrier), and one time dimension, when the channels are assumed to be, respectively, time slot or frame depending.
- With most relaying systems, the information symbols to transmit form symbol matrices $S\in C^{N\times R}$ containing *R* data streams composed of *N* symbols each. However, in the case of OFDM systems, they can form third-order tensors $S\in C^{N\times R\times F}$, where *F* is the number of sub-carriers employed, as in [47].
- Depending on the transmission strategy used (in terms of time spreading) and the structure of the relay system, the transmission process is divided into several phases. For instance, in a one-way two-hop communication system, transmission may be performed in *P* time-blocks, each consisting of *N* symbol periods. Time repetition induces redundancy in the transmitted symbols, i.e., time diversity via coding.

## 4. Overview of Codings Used in Cooperative Systems

In Table 10, we summarize the main codings used in cooperative systems: Khatri-Rao space-time (KRST), simplified KRST (SKRST), double KRSTF (DKRSTF), tensor space-time-frequency (TSTF), simplified TSTF (STSTF), tensor space-time (TST), multiple symbol matrices Kronecker product (MSMKron), multiple symbol matrices Khatri-Rao product (MSMKR), and combined SKRST-MSMKR and TST-MSMKron codings.

Below, we make some comments on the codings considered in Table 10.

- The dimensions (M,P,J,F) represent the numbers of transmit antennas, transmission blocks, time slots or chips, and sub-carriers, respectively. In the case of a single symbol matrix $S\in C^{N\times R}$, *R* is the number of data streams, and *N* is the number of symbols per data stream. When *Q* symbol matrices $S^{\left(q\right)}\in C^{N_{q}\times R_{q}}$ are considered, $R_{q}$ and $N_{q}$ represent the numbers of data streams and symbols per data stream in the *q*th symbol matrix $S^{\left(q\right)}$, respectively, with q∈〈Q〉.
- With the KRST coding [50], pre- and post-coding matrices $\left(C\in C^{M\times M},W\in C^{P\times M}\right)$ are used for encoding the information symbols contained in the symbol matrix $S\in C^{M\times R}$. The pre-coding one linearly combines the *M* symbols of each data stream $s_{.r}$ to deliver the matrix of pre-coded signals $V=S^{T}\;C\in C^{R\times M}$ which are then spread over *P* slots using the post-coding matrix W to give the third-order tensor $U\in C^{R\times M\times P}$ of encoded signals, defined as:
  (13) $$U=V\underset{m}{⊙}W=S^{T}\;C\;\underset{m}{⊙}\;W.$$
  In scalar form, we have: $v_{r,m}=\sum_{l=1}^{M}s_{l,r}\;c_{l,m}$ and $u_{r,m,p}=v_{r,m}w_{p,m}=\sum_{l=1}^{M}s_{l,r}\;c_{l,m}w_{p,m}$. A tall mode-2 matrix unfolding of the coded signals tensor is given by $U_{RP\times M}=V⋄W=S^{T}\;C⋄W$. This writing highlights the Khatri–Rao product of the pre-coded signals matrix V with the post-coding matrix W, which justifies the KRST name of this coding.
- A simplified version of the KRST coding, denoted SKRST, was introduced in [21,22] for designing tensor-based two-hop communication systems. This coding consists of a simple Khatri-Rao product $U=C⋄S\in C^{PN\times M}$ between a coding matrix $C\in C^{P\times M}$ and a symbol matrix $S\in C^{N\times M}$, where *P* is the code length. This coding introduces time spreading of symbols.
  The matrix U of coded signals can be transformed into a third-order tensor $U\in C^{M\times P\times N}$, which satisfies the CPD model $\left[[I_{M},C,S;M]\right]$, such as:
  (14) $$u_{m,p,n}=\sum_{q=1}^{M}\delta_{m,q}c_{p,q}s_{n,q}=c_{p,m}s_{n,m}.$$
  It should be noted that, contrary to SKRST coding, KRST coding does not impose that the number *R* of data streams be equal to the number *M* of transmit antennas.
- The DKRSTF coding [51] can be viewed as an OFDM extension of the SKRST one delivering a fourth-order tensor $U\in C^{F\times N\times M\times P}$ for the coded signals given by $u_{f,n,m,p}=v_{f,n,m}w_{p,m}=\sum_{l=1}^{M}a_{f,l}s_{n,l}c_{l,m}w_{p,m}$.
  The third-order tensor $V\in C^{F\times N\times M}$, which contains the space–frequency pre-coded signals, satisfies the CPD model $\left[[A,S,C^{T};M]\right]$ and is as such: $V_{FN\times M}=\left(A⋄S\right)C$, where the matrices $C\in C^{M\times M}$ and $A\in C^{F\times M}$ are associated with the space-frequency pre-coding, whereas $W\in C^{P\times M}$ is the time post-coding matrix. The space–time–frequency coded signals tensor can also be written as
  (15) $$U=V_{FN\times M}\underset{m}{⊙}W=\left(A⋄S\right)C\;\underset{m}{⊙}\;W\in C^{F\times N\times M\times P},$$
  which gives the following matrix unfolding: $U_{FNP\times M}=V_{FN\times M}⋄W=\left(A⋄S\right)C⋄W$. This expression highlights the double Khatri–Rao STF (DKRSTF) coding, one corresponding to a space–frequency pre-coding by means of the matrices (A,C), whereas the other one corresponds to a time post-coding provided by the matrix W. The DKRSTF coding is an extension of the SKRST one.
- The TSTF coding provides a fifth-order tensor of coded signals [15]. It can be viewed as an extension of the TST coding [52] for an OFDM system with a multicarrier transmission, which allows a supplementary spread of the information symbols in the frequency and chip (or time slot) domains.
- Note that the KRST/TST, DKRSTF, and TSTF codings provide third-, fourth- and fifth-order tensors U of coded signals, respectively, inducing a greater diversity gain for the TSTF coding in comparison with the other codings [11].
  A drawback shared by SKRST and DKRSTF codings concerns the constraint that the number of data streams must be equal to the number of transmit antennas (R=M), while for the KRST coding, this constraint relates to the number of symbols per data stream (N=M). That is not the case of tensor codings (TST and TSTF). See the dimensions of the symbol matrix S in Table 10.
- Multiple Kronecker and Khatri–Rao products of symbol matrices, denoted MSMKron and MSMKR, can be viewed as extensions of the KRST coding [50] and as simplified versions of the MKronST and MKRST codings proposed in [25] without a precoding matrix.
  With these codings, each symbol $s_{i,j}^{\left(q\right)}$ of a given symbol matrix $S^{\left(q\right)}$ is duplicated at the transmission via the Khatri–Rao and Kronecker products of $S^{\left(q\right)}$ with the other symbol matrices $S^{\left(q^{\prime }\right)}$, $q^{\prime }\neq q$.
  These multiple KR and Kron products induce a mutual ST spreading of transmitted symbols and therefore an extra ST diversity. Note that efficient decoding methods based on rank-one matrix/tensor approximations can be found in [11,13,25,33] for recovering each individual symbol matrix from an estimated KR or Kron product of multiple symbol matrices.
- Combining MSMKR and MSMKron with SKRST and TST codings gave rise to the SKRST-MSMKR and TST-MSMKron codings, respectively, proposed for the first time in [53,54].

## 5. Overview of Two-Hop Systems

In this section, we present several two-hop systems in an unified way. These systems are composed of a source (S) which sends information symbols to a destination (D), via a relay (R) or an IRS (I), as illustrated in Figure 3a,g. The multi-relay case of Figure 3d is also considered.

With relay-assisted systems, the tensor-based approach allows deriving semi-blind receivers for joint symbol and channel estimation, which depend on different codings.

The source and destination nodes are equipped with $M_{S}$ and $M_{D}$ antennas, respectively, whereas the relay uses $M_{R}$ antennas for reception and $M_{T}$ for transmission.

The IRS is assumed to be composed of $M_{I}$ identical unit cells, which create attenuation and phase shifts on the reflected signals, considered time-varying at each time slot *p* and modeled by means of a matrix $G\in C^{P\times M_{I}}$. Each row $g_{p.}$ contains the amplitude and phase shift coefficients associated with the perturbations introduced by the $M_{I}$ cells of the IRS, at the time slot *p*.

The channels between the source and relay (SR) or IRS (SI) are modeled by means of matrices $H^{\left(SR\right)}\in C^{M_{R}\times M_{S}}$ and $H^{\left(SI\right)}\in C^{M_{I}\times M_{S}}$. Similarly, the channels between the relay or IRS and the destination are denoted $H^{\left(RD\right)}\in C^{M_{D}\times M_{T}}$ and $H^{\left(ID\right)}\in C^{M_{D}\times M_{I}}$.

The considered relay- and IRS-assisted systems are listed below:

- Relay-assisted two-hop system using SKRST codings at the source and relay nodes, with AF protocol; see Table 11;
- Relay-assisted two-hop system using SKRST-MSMKR codings at the source and relay nodes, with DF protocol and time-varying multipath channel; see Table 12;
- Relay-assisted two-hop system using third-order TST codings at the source and relay nodes, with AF and DF protocols; see Table 13; an extension of the multi-user case is also considered, illustrated by means of Figure 8.
- Multi-relay-assisted system using third-order TST codings at the source and relay nodes, with AF protocol and *B* relays in parallel and with each relay equipped with a different TST coding; see Table 14;
- IRS-assisted system using SKRST coding at the source; see Table 15.

Note that the direct link between the source and the destination nodes is assumed to be unavailable. Moreover, for simplifying the presentation, the noiseless case is considered.

### 5.1. Relay Two-Hop System Using SKRST Codings

Equations of the system using SKRST coding matrices $C^{\left(S\right)}\in C^{P\times M_{S}}$ and $C^{\left(R\right)}\in C^{J\times M_{R}}$ at the source and relay, respectively, without decoding at relay, are summarized in Table 11. These matrix equations can be reformulated using the tensor formalism as follows.

**Table 11:** Two-hop systems with SKRST codings.

Ref./Signals	Symbols/Codings	Channels	Encoded/Received Signals	Dimensions
[22]	$S\in C^{N\times M_{S}}$			
		**First hop**		
Signals coded at source	$C^{\left(S\right)}\in C^{P\times M_{S}}$		$U_{PN\times M_{S}}^{\left(S\right)}=C^{\left(S\right)}⋄S$	$PN\times M_{S}$
Signals received at relay		$H^{\left(SR\right)}\in C^{M_{R}\times M_{S}}$	$X_{M_{R}\times PN}^{\left(R\right)}=H^{\left(SR\right)}U_{M_{S}\times PN}^{\left(S\right)}$	$M_{R}\times PN$
			$=H^{\left(SR\right)}{\left(C^{\left(S\right)}⋄S\right)}^{T}$	
		**Second hop**		
Signals coded at relay	$C^{\left(R\right)}\in C^{J\times M_{R}}$		$U_{JPN\times M_{R}}^{\left(R\right)}=C^{\left(R\right)}⋄X_{PN\times M_{R}}^{\left(R\right)}$	$JPN\times M_{R}$
			$=C^{\left(R\right)}⋄\left(C^{\left(S\right)}⋄S\right){\left(H^{\left(SR\right)}\right)}^{T}$	
Signals received at destination		$H^{\left(RD\right)}\in C^{M_{D}\times M_{R}}$	$X_{M_{D}\times JPN}^{\left(D\right)}=H^{\left(RD\right)}U_{M_{R}\times JPN}^{\left(R\right)}$	$M_{D}\times JPN$
			$=H^{\left(RD\right)}{\left(C^{\left(R\right)}⋄X_{PN\times M_{R}}^{\left(R\right)}\right)}^{T}$	
			$=H^{\left(RD\right)}{\left(C^{\left(R\right)}⋄\left(C^{\left(S\right)}⋄S\right){\left(H^{\left(SR\right)}\right)}^{T}\right)}^{T}$	

As shown in Equation (14), the Khatri–Rao products defining the matrices $U_{PN\times M_{S}}^{\left(S\right)}$ and $U_{JPN\times M_{R}}^{\left(R\right)}$ of signals coded at source and relay can be associated with the third-order tensors $U^{\left(S\right)}\in C^{M_{S}\times P\times N}$ and $U_{c}^{\left(R\right)}\in C^{M_{R}\times J\times PN}$, which satisfy the following CPD models:

(16) $$\begin{array}{cc} U^{\left(S\right)}= & \;\;I_{M_{S}}\times_{1}I_{M_{S}}\times_{2}C^{\left(S\right)}\times_{3}S \end{array}$$

(17) $$\begin{array}{cc} U_{c}^{\left(R\right)}= & \;\;I_{M_{R}}\times_{1}I_{M_{R}}\times_{2}C^{\left(R\right)}\times_{3}X_{PN\times M_{R}}^{\left(R\right)}. \end{array}$$

The matrices $X_{M_{R}\times PN}^{\left(R\right)}$ and $X_{M_{D}\times JPN}^{\left(D\right)}$ containing the signals received at relay and destination can also be associated with third-order tensors $X^{\left(R\right)}\in C^{M_{R}\times P\times N}$ and $X_{c}^{\left(D\right)}\in C^{M_{D}\times J\times PN}$, which satisfy CPD models respectively deduced from Equations (16) and (17) as follows:

(18) $$\begin{array}{cc} X^{\left(R\right)}= & \;\;U^{\left(S\right)}\times_{1}H^{\left(SR\right)}\;\;⟺\;\;X^{\left(R\right)}=I_{M_{S}}\times_{1}H^{\left(SR\right)}\times_{2}C^{\left(S\right)}\times_{3}S \end{array}$$

(19) $$\begin{array}{cc} X_{c}^{\left(D\right)}= & \;\;U_{c}^{\left(R\right)}\times_{1}H^{\left(RD\right)}\;\;⟺\;\;X_{c}^{\left(D\right)}=I_{M_{R}}\times_{1}H^{\left(RD\right)}\times_{2}C^{\left(R\right)}\times_{3}X_{PN\times M_{R}}^{\left(R\right)}. \end{array}$$

Note that $U_{c}^{\left(R\right)}$ and $X_{c}^{\left(D\right)}$ are contracted forms of fourth-order tensors $U^{\left(R\right)}\in C^{M_{R}\times J\times P\times N}$ and $X^{\left(D\right)}\in C^{M_{D}\times J\times P\times N}$ resulting from a combination of third and fourth modes (*p* and *n*).

The contracted tensor $X_{c}^{\left(D\right)}$ satisfies a CPD model whose third matrix factor is a matrix unfolding $X_{PN\times M_{R}}^{\left(R\right)}$ of the tensor $X^{\left(R\right)}$, which satisfies itself a CPD model.

From the CPD models (18) and (19) of tensors $X^{\left(R\right)}$ and $X_{c}^{\left(D\right)}$, with the matrix unfolding $X_{PN\times M_{R}}^{\left(R\right)}$ replaced by the scalar entry $x_{m_{R},p,n}^{\left(R\right)}$ we deduce the following equations:

(20) $$\begin{array}{cc} x_{m_{R},p,n}^{\left(R\right)}= & \;\sum_{m_{S}}h_{m_{R},m_{S}}^{\left(SR\right)}c_{p,m_{S}}^{\left(S\right)}s_{n,m_{S}} \end{array}$$

(21) $$\begin{array}{cc} x_{m_{D},j,p,n}^{\left(D\right)}= & \;\sum_{m_{R}}h_{m_{D},m_{R}}^{\left(RD\right)}c_{j,m_{R}}^{\left(R\right)}x_{m_{R},p,n}^{\left(R\right)}. \end{array}$$

Replacing $x_{m_{R},p,n}^{\left(R\right)}$ by its expression (20) into (21), the signal received at destination by the $m_{D}$-th antenna associated with the *n*-th symbol period of the *p*-th time-block (at the source) and *j*-th time block (at the relay), is given by:

(22) $$x_{m_{D},j,p,n}^{\left(D\right)}=\sum_{m_{R}}\sum_{m_{S}}h_{m_{D},m_{R}}^{\left(RD\right)}c_{j,m_{R}}^{\left(R\right)}h_{m_{R},m_{S}}^{\left(SR\right)}c_{p,m_{S}}^{\left(S\right)}s_{n,m_{S}}.$$

This equation corresponds to a nested CPD model [22,51], i.e., a nesting of two CPD models that share a common matrix factor, for the fourth-order tensor $X^{\left(D\right)}$ of signals received at destination. In (22), the blue color is associated with the sum over index $m_{S}$ due to the CPD model (18) of the tensor $X^{\left(R\right)}$, while the red color is used for the sum over index $m_{R}$ associated with the CPD model $\left[[H^{\left(RD\right)},C^{\left(R\right)},{\left(H^{\left(SR\right)}\right)}^{T};M_{R}]\right]$ of the effective channel tensor $H\in C^{M_{D}\times J\times M_{S}}$ between the source and the destination nodes, defined as follows:

(23) $$h_{m_{D},j,m_{S}}=\sum_{m_{R}}h_{m_{D},m_{R}}^{\left(RD\right)}c_{j,m_{R}}^{\left(R\right)}h_{m_{R},m_{S}}^{\left(SR\right)}\;\;⟺\;\;H=I_{M_{R}}\times_{1}H^{\left(RD\right)}\times_{2}C^{\left(R\right)}\times_{3}{\left(H^{\left(SR\right)}\right)}^{T}.$$

**Figure 4:**
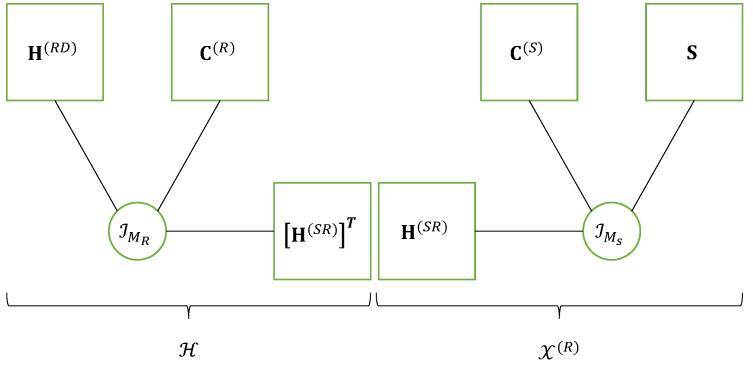
Nested CPD model of a relaying system using SKRST codings.

The matrix factor $H^{\left(SR\right)}$ shared by the CPD models of H and $X^{\left(R\right)}$, is in green. The nested CPD model of the tensor $X^{\left(D\right)}$ of signals received at destination is illustrated by means of Figure 4, highlighting the CPD models of the tensors H and $X^{\left(R\right)}$. An extension of the multi-hop case is proposed in [28].

**Remark** **4.** *If we choose J=N, Equation (22) becomes:*

(24) $$x_{m_{D},p,n}^{\left(D\right)}=\sum_{m_{R}}\sum_{m_{S}}h_{m_{D},m_{R}}^{\left(RD\right)}c_{n,m_{R}}^{\left(R\right)}h_{m_{R},m_{S}}^{\left(SR\right)}c_{p,m_{S}}^{\left(S\right)}s_{n,m_{S}}.$$

*In this case, $X^{\left(D\right)}\in C^{M_{D}\times P\times N}$ is a third-order tensor that satisfies a PARATUCK model [21]. The system then benefits from three diversities associated with the three dimensions of $X^{D)}$, corresponding to space $\left(M_{D}\right)$, source code (P), and time (N) diversities. Compared with the relaying system presented in Table 11, we conclude that the double SKRST coding at source and relay (with J≠N) allows us to introduce an additional relay code diversity (J), leading to a nested CPD model for the fourth-order tensor $X^{\left(D\right)}$ described by Equation (22). This increase in diversity is at the origin of a SER performance improvement, as illustrated in [22].*

### 5.2. Relay Two-Hop System Using SKRST-MSMKR Codings, with DF Protocol and Time-Varying Multipath Channel

In this section, we consider a MIMO relaying system equipped with uniform linear arrays (ULAs) at each node, which uses SKRST-MSMKR codings at the source and relay nodes. The transmission is composed of *T* blocks, meaning that each symbol matrix is transmitted *T* times. The DF protocol, employed at the relay, consists of estimating the information symbols and then re-encoding the estimated symbols before their transmission towards the destination node. In the following, we first define the SKRST-MSMKR coding at the source. Then, the source–relay channel will be described as a third-order tensor $H^{\left(SR\right)}\in C^{M_{R}\times T\times M_{S}}$, which is time-dependent and satisfies a CPD model. Finally, the signals received at the relay and destination nodes will be presented under the form of two fourth-order tensors satisfying nested CPD and cascaded nested CPD models, respectively.

#### 5.2.1. SKRST-MSMKR Coding

The information symbols are coded at the source using the coding matrix $C^{\left(S\right)}\in C^{P\times M_{S}}$ combined with MSMKR, which gives the following coded signals matrix:

(25) $$U_{PN\times M_{S}}^{\left(S\right)}=C^{\left(S\right)}⋄S=C^{\left(S\right)}⋄S^{\left(1\right)}⋄\ldots ⋄S^{\left(Q\right)},$$

where $S=\overset{Q}{\underset{q=1}{⋄}}S^{\left(q\right)}≜S^{\left(1\right)}⋄\ldots ⋄S^{\left(Q\right)}\in C^{N\times M_{S}}$, with $S^{\left(q\right)}\in C^{N_{q}\times M_{S}}$ for q∈〈Q〉, and $N=\prod_{q=1}^{Q}N_{q}$, which gives the following third-order contracted tensor $U_{c}^{\left(S\right)}\in C^{M_{S}\times P\times N}$ of coded signals:

(26) $$u_{m_{S},p,n}^{\left(S\right)}=c_{p,m_{S}}^{\left(S\right)}s_{n,m_{S}}\;\;⟺\;\;U_{c}^{\left(S\right)}=I_{M_{S}}\times_{1}I_{M_{S}}\times_{2}C^{\left(S\right)}\times_{3}S.$$

Replacing S by its MSMKR expression leads to the following developed form of the coded signals tensor $U^{\left(S\right)}\in C^{M_{S}\times P\times N_{1}\times \cdots \times N_{Q}}$:

(27) $$u_{m_{S},p,n_{1},\cdots ,n_{Q}}^{\left(S\right)}=c_{p,m_{S}}^{\left(S\right)}\prod_{q=1}^{Q}s_{n_{q},m_{S}}^{\left(q\right)}\;\;⟺\;\;U^{\left(S\right)}=I_{M_{S}}\times_{1}I_{M_{S}}\times_{2}C^{\left(S\right)}\times_{3}S^{\left(1\right)}\cdots \times_{Q+2}S^{\left(Q\right)}.$$

#### 5.2.2. Channel Modeling

The channel between the source and the relay nodes is assumed to be characterized by *L* paths, DoD and DoA angles $\left(\phi_{l},\theta_{l}\right)$, with l∈〈L〉, and fading coefficients $w_{t,l}$ which depend on the transmission block *t* and path *l*. The steering matrices $A^{\left(S\right)}$ and $A^{\left(R\right)}$ at source and relay, respectively, are given by:

(28) $$\begin{array}{cc} A^{\left(S\right)}= & \;\left[a^{\left(S\right)}\left(\phi_{1}\right),\cdots ,a^{\left(S\right)}\left(\phi_{L}\right)\right]\in C^{M_{S}\times L} \end{array}$$

(29) $$\begin{array}{cc} A^{\left(R\right)}= & \;\left[a^{\left(R\right)}\left(\theta_{1}\right),\cdots ,a^{\left(R\right)}\left(\theta_{L}\right)\right]\in C^{M_{R}\times L}, \end{array}$$

with $\left(i^{2}=-1\right)$:

(30) $$\begin{array}{cc} a^{\left(S\right)}\left(\phi_{l}\right)= & \;{\left[1,e^{-i\pi \;\sin(\phi_{l})},\cdots ,e^{-i\pi \;\left(M_{S}-1\right)\;\sin\left(\phi_{l}\right)}\right]}^{T}\in C^{M_{S}} \end{array}$$

(31) $$\begin{array}{cc} a^{\left(R\right)}\left(\theta_{l}\right)= & \;{\left[1,e^{-i\pi \;\sin(\theta_{l})},\cdots ,e^{-i\pi \;\left(M_{S}-1\right)\;\sin\left(\theta_{l}\right)}\right]}^{T}\in C^{M_{R}}. \end{array}$$

The matrix of fading coefficients is formed by *T* rows $w_{t}^{T}\in C^{1\times L}$, t∈〈T〉, written as:

(32) $$W=\left[\begin{array}{c} w_{1}^{T}\\ \vdots \\ w_{T}^{T} \end{array}\right]\in C^{T\times L}.$$

Note that the DoD and DoA angles are assumed to be constant during *T* blocks. The channel between source and relay nodes during the time block *t* satisfies the following equation:

(33) $$h_{m_{R},t,m_{S}}^{\left(SR\right)}=\sum_{l=1}^{L}a_{m_{R},l}^{\left(R\right)}w_{t,l}a_{m_{S},l}^{\left(S\right)}\;\;⟺\;\;H_{M_{R}\times M_{S}}^{\left(SR\right)}\left(t\right)=A^{\left(R\right)}D_{t}\left(W\right){\left[A^{\left(S\right)}\right]}^{T}\;\;\;,\;\;\;t\in \langle T\rangle ,$$

with $D_{t}\left(W\right)=\mathrm{diag}\left(w_{t}\right)$. Equation (33) can be interpreted as the *t*-th lateral slice of the third-order channel tensor $H^{\left(SR\right)}\in C^{M_{R}\times T\times M_{S}}$, which satisfies the rank-*L* CPD model $\left[[A^{\left(R\right)},W,A^{\left(S\right)};L]\right]$, whose tall mode-3 unfolding is given by:

(34) $$H_{TM_{R}\times M_{S}}^{\left(SR\right)}=\left(W⋄A^{\left(R\right)}\right){\left(A^{\left(S\right)}\right)}^{T}\in C^{TM_{R}\times M_{S}}.$$

The coded signals (27) are transmitted by the $M_{S}$ antennas of the source through the *l*-th path according to the following equation:

(35) $$t_{l,p,n_{1},\cdots ,n_{Q}}^{\left(S\right)}=\sum_{m_{S}=1}^{M_{S}}a_{m_{S},l}^{\left(S\right)}u_{p,n_{1},\cdots ,n_{Q},m_{S}}^{\left(S\right)}=\sum_{m_{S}=1}^{M_{S}}a_{m_{S},l}^{\left(S\right)}c_{p,m_{S}}^{\left(S\right)}\prod_{q=1}^{Q}s_{n_{q},m_{S}}^{\left(q\right)}.$$

Using the contracted form (26) of coded signals allows us to rewrite the tensor $T^{\left(S\right)}\in C^{L\times P\times N_{1}\times \cdots \times N_{Q}}$ in a contracted form $T_{c}^{\left(S\right)}\in C^{L\times P\times N}$ such as:

(36) $$t_{l,p,n}^{\left(S\right)}=\sum_{m_{S}=1}^{M_{S}}a_{m_{S},l}^{\left(S\right)}u_{p,n,m_{S}}^{\left(S\right)}=\sum_{m_{S}=1}^{M_{S}}a_{m_{S},l}^{\left(S\right)}c_{p,m_{S}}^{\left(S\right)}s_{n,m_{S}}.$$

This equation corresponds to the CPD model $\left[[{\left(A^{\left(S\right)}\right)}^{T},C^{\left(S\right)},S;M_{S}]\right]$ of the contracted tensor $T_{c}^{\left(S\right)}$ of symbols transmitted at the source, obtained by combining the last *Q* modes. From this CPD model, we deduce the following flat mode-1 unfolding of $T_{c}^{\left(S\right)}$:

(37) $$T_{L\times PN}^{\left(S\right)}={\left(A^{\left(S\right)}\right)}^{T}{\left(C^{\left(S\right)}⋄S\right)}^{T}.$$

#### 5.2.3. Signals Received at the Relay

The signals received at relay during *T* blocks result from the transmission of the coded signals tensor $U^{\left(S\right)}$ through the channel tensor $H^{\left(SR\right)}$ via the following matrix equation:

(38) $$X_{TM_{R}\times PN}^{\left(R\right)}=H_{TM_{R}\times M_{S}}^{\left(SR\right)}U_{M_{S}\times PN}^{\left(S\right)}.$$

Replacing $H_{TM_{R}\times M_{S}}^{\left(SR\right)}$ and $U_{M_{S}\times PN}^{\left(S\right)}$ by their expressions (34) and (25) gives:

(39) $$X_{TM_{R}\times PN}^{\left(R\right)}=\left(W⋄A^{\left(R\right)}\right){\left(A^{\left(S\right)}\right)}^{T}{\left(C^{\left(S\right)}⋄S\right)}^{T}.$$

**Figure 5:**
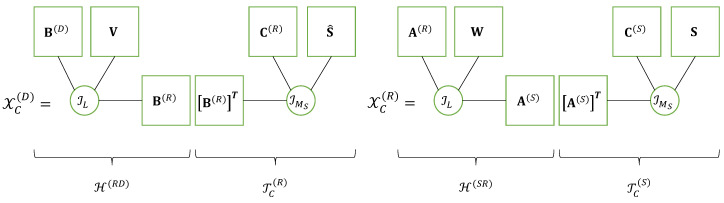
Cascaded nested CPD model of a time-varying two-hop multipath system using DF protocol and SKRST-MSMKR codings.

This equation highlights the nesting of the CPD model of the channel tensor $H^{\left(SR\right)}$ (in red color) with the CPD model of the transmitted signals contracted tensor $T_{c}^{\left(S\right)}$ (in blue color), where the matrix factor $A^{\left(S\right)}$ (in green) is shared by both CPD models. The nested CPD model of the received signal’s contracted tensor $X_{c}^{\left(R\right)}\in C^{M_{R}\times T\times P\times N}$ is shown on the right part of Figure 5.

The nested CPD model (39) of $X_{c}^{\left(R\right)}$ can also be interpreted as the contraction between the channel tensor $H^{\left(SR\right)}$ and the contracted tensor $U_{c}^{\left(S\right)}$ of coded signals, along their common mode $m_{S}$; that means:

(40) $$X_{c}^{\left(D\right)}=H^{\left(SR\right)}\times_{3}^{1}U_{c}^{\left(S\right)}.$$

#### 5.2.4. Signals Received at Destination

Due to the DF protocol used at relay, equations of both hops are similar, with the following correspondences:

(41) $$\begin{array}{cc} (C^{\left(S\right)},A^{\left(S\right)},A^{\left(R\right)},W,S,H^{\left(SR\right)})\;\;⟷ & \;\;(C^{\left(R\right)},B^{\left(R\right)},B^{\left(D\right)},V,\hat{S},H^{\left(RD\right)}) \end{array}$$

(42) $$\begin{array}{cc} (M_{S},M_{R},P)\;\;⟷ & \;\;(M_{S},M_{D},J). \end{array}$$

Using these correspondences (41), Equation (39) becomes the signal received at destination:

(43) $$X_{TM_{D}\times JN}^{\left(D\right)}=\left(V⋄B^{\left(D\right)}\right){\left(B^{\left(R\right)}\right)}^{T}{\left(C^{\left(R\right)}⋄\hat{S}\right)}^{T}.$$

We conclude that the contracted tensor $X_{c}^{\left(D\right)}$ of signals received at destination satisfies a new cascaded nested CPD model, as shown in Figure 5. This system constitutes a two-hop extension of the point-to-point system presented in [53].

In Table 12, we summarize the matrix equations of the relaying two-hop system using SKRST-MSMKR codings, with DF protocol and time-varying multipath channel.

Note that, differently from the relaying system presented in Table 11, the signals encoded at relay are now the symbol matrices ($\hat{S}=\overset{Q}{\underset{q=1}{⋄}}{\hat{S}}^{\left(q\right)}$) estimated at relay, and not the signals received at relay. That implies the numbers of transmit antennas at source and relay must be equal $\left(M_{T}=M_{S}\right)$.

It is worth noting that comparing Figure 4 and Figure 5 highlights the following correspondences between these two relaying systems. For the first hop, we have:

(44) $$\left(S,C^{\left(S\right)},H^{\left(SR\right)},C^{\left(R\right)},H^{\left(RD\right)}\right)\;⟺\;\left(S,C^{\left(S\right)},{\left(A^{\left(S\right)}\right)}^{T},W,A^{\left(R\right)}\right)$$

and for the second hop:

(45) $$\left(S,C^{\left(S\right)},H^{\left(SR\right)},C^{\left(R\right)},H^{\left(RD\right)}\right)\;⟺\;\left(\hat{S},C^{\left(R\right)},{\left(B^{\left(R\right)}\right)}^{T},V,B^{\left(D\right)}\right)$$

that means the channel matrices ($H^{\left(SR\right)},H^{\left(RD\right)}$) are replaced by the steering matrices (${\left(A^{\left(S\right)}\right)}^{T},A^{\left(R\right)}$) for the first hop, and (${\left(B^{\left(R\right)}\right)}^{T},B^{\left(D\right)}$) for the second hop, whereas the coding matrices ($C^{\left(S\right)},C^{\left(R\right)}$) are respectively replaced by ($C^{\left(S\right)},W$) and ($C^{\left(R\right)},V$).

**Table 12:** Two-hop systems with SKRST-MSMKR codings and DF protocol.

Signals	Symbols/Codings	Channels	Encoded/Received Signals	Dimensions
	$S=\overset{Q}{\underset{q=1}{⋄}}S^{\left(q\right)}\in C^{N\times M_{S}}$			
	$S^{\left(q\right)}\in C^{N_{q}\times M_{S}}$ for q∈〈Q〉			
		**First hop**		
Signals coded at source	$C^{\left(S\right)}\in C^{P\times M_{S}}$		$U_{PN\times M_{S}}^{\left(S\right)}=C^{\left(S\right)}⋄S=C^{\left(S\right)}⋄S^{\left(1\right)}⋄S^{\left(Q\right)}$	$PN\times M_{S}$
Signals transmitted by source		$A^{\left(S\right)}\in C^{M_{S}\times L}$	$T_{L\times PN}^{\left(S\right)}={\left(A^{\left(S\right)}\right)}^{T}{\left(C^{\left(S\right)}⋄S\right)}^{T}$	L×PN
Signals received at relay		$A^{\left(R\right)}\in C^{M_{R}\times L}$	$X_{TM_{R}\times PN}^{\left(R\right)}=H_{TM_{R}\times M_{S}}^{\left(SR\right)}U_{M_{S}\times PN}^{\left(S\right)}$	$TM_{R}\times PN$
		$W\in C^{T\times L}$	$=\left(W⋄A^{\left(R\right)}\right){\left(A^{\left(S\right)}\right)}^{T}{\left(C^{\left(S\right)}⋄S\right)}^{T}$	
		**Second hop**		
Signals coded at relay	$C^{\left(R\right)}\in C^{J\times M_{S}}$		$U_{JN\times M_{S}}^{\left(R\right)}=C^{\left(R\right)}⋄\hat{S}$	$JN\times M_{S}$
Signals transmitted by relay		$B^{\left(R\right)}\in C^{M_{S}\times L}$	$T_{L\times JN}^{\left(R\right)}={\left(B^{\left(R\right)}\right)}^{T}{\left(C^{\left(R\right)}⋄\hat{S}\right)}^{T}$	L×JN
Signals received at destination		$B^{\left(D\right)}\in C^{M_{D}\times L}$	$X_{TM_{D}\times JN}^{\left(D\right)}=H_{TM_{D}\times M_{S}}^{\left(RD\right)}U_{M_{S}\times JN}^{\left(R\right)}$	$TM_{D}\times JN$
		$V\in C^{T\times L}$	$=\left(V⋄B^{\left(D\right)}\right){\left(B^{\left(R\right)}\right)}^{T}{\left(C^{\left(R\right)}⋄\hat{S}\right)}^{T}$

**Remark** **5.** *Semi-blind receivers can be developed to jointly estimate the individual channels and the multiple Kronecker S of the symbol matrices. Then, in a second stage, a closed-form algorithm called the Kronecker factorization (KronF) algorithm is used to separate the symbol matrices. Such receivers will be presented in a companion paper.*

### 5.3. Relay Two-Hop Systems Using TST Codings

In Table 13, we summarize the equations of the two-hop system proposed in [24] using a third-order TST coding at both the source and relay, with the AF protocol at the relay, i.e., without decoding at the relay.

**Table 13:** Two-hop systems with TST codings.

Ref./Signals	Symbols/Codings	Channels	Encoded/Received Signals	Dimensions
[24]	$S\in C^{N\times R}$			
		**First hop**		
Signals coded at source	$C^{\left(S\right)}\in C^{M_{S}\times P\times R}$		$U^{\left(S\right)}=C^{\left(S\right)}\times_{3}S$	$M_{S}\times P\times N$
Signals received at relay		$H^{\left(SR\right)}\in C^{M_{R}\times M_{S}}$	$X^{\left(R\right)}=U^{\left(S\right)}\times_{1}H^{\left(SR\right)}$	$M_{R}\times P\times N$
		**Second hop**		
Signals coded at relay	$C^{\left(R\right)}\in C^{M_{T}\times J\times M_{R}}$		$U^{\left(R\right)}=C^{\left(R\right)}\times_{3}^{1}X^{\left(R\right)}$	$M_{T}\times J\times P\times N$
Signals received at destination		$H^{\left(RD\right)}\in C^{M_{D}\times M_{T}}$	$X^{\left(D\right)}=U^{\left(R\right)}\times_{1}H^{\left(RD\right)}$	$M_{D}\times J\times P\times N$

From the equations in Table 13, we can write the tensor $X^{\left(D\right)}\in C^{M_{D}\times J\times P\times N}$ of signals received at destination as:

(46) $$X^{\left(D\right)}=U^{\left(R\right)}\times_{1}H^{\left(RD\right)}=C^{\left(R\right)}\times_{1}H^{\left(RD\right)}\times_{3}^{1}X^{\left(R\right)},$$

with:

(47) $$X^{\left(R\right)}=C^{\left(S\right)}\times_{1}H^{\left(SR\right)}\times_{3}S\in C^{M_{R}\times P\times N}.$$

Noting that the third-order tensor $C^{\left(R\right)}\times_{1}H^{\left(RD\right)}\in C^{M_{D}\times J\times M_{R}}$ satisfies the Tucker-(1,3) model $\left[[C^{\left(R\right)};H^{\left(RD\right)},I_{J},I_{M_{R}}]\right]$ and the tensor $X^{\left(R\right)}$ satisfies the Tucker-(2,3) model $\left[[C^{\left(S\right)};H^{\left(SR\right)},I_{P},S]\right]$, Equation (46) can be interpreted as a contraction operation, denoted $\times_{3}^{1}$, along the common mode $m_{R}$ of these two tensors.

Equations (46) and (47) can also be written as:

(48) $$\begin{array}{cc} X^{\left(D\right)}= & \;H^{\left(RD\right)}\times_{2}^{1}C^{\left(R\right)}\times_{3}^{1}X^{\left(R\right)} \end{array}$$

(49) $$\begin{array}{cc} X^{\left(R\right)}= & \;H^{\left(SR\right)}\times_{2}^{1}C^{\left(S\right)}\times_{3}^{2}S. \end{array}$$

Combining these equations leads to the following expression for the tensor $X^{\left(D\right)}$:

(50) $$X^{\left(D\right)}=H^{\left(RD\right)}\times_{2}^{1}C^{\left(R\right)}\times_{3}^{1}H^{\left(SR\right)}\times_{2}^{1}C^{\left(S\right)}\times_{3}^{2}S.$$

**Figure 6:**
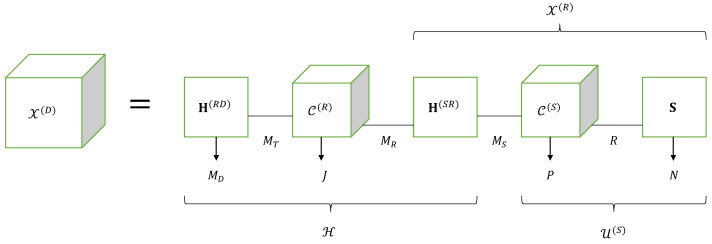
Tucker train model of a relaying system using TST codings.

This writing highlights the Tucker train model of $X^{\left(D\right)}$, illustrated by means of Figure 6.

Now, let us define the effective channel tensor $H\in C^{M_{D}\times J\times M_{S}}$ between the source and destination nodes as:

(51) $$H=H^{\left(RD\right)}\times_{2}^{1}C^{\left(R\right)}\times_{3}^{1}H^{\left(SR\right)}=C^{\left(R\right)}\times_{1}H^{\left(RD\right)}\times_{3}{\left(H^{\left(SR\right)}\right)}^{T}.$$

This tensor satisfies the Tucker-(2,3) model $\left[[C^{\left(R\right)};H^{\left(RD\right)},I_{J},{\left(H^{\left(SR\right)}\right)}^{T}]\right]$, with the following scalar expression:

(52) $$h_{m_{D},j,m_{S}}=\sum_{m_{T}}\sum_{m_{R}}c_{m_{T},j,m_{R}}^{\left(R\right)}h_{m_{D},m_{T}}^{\left(RD\right)}h_{m_{R},m_{S}}^{\left(SR\right)}.$$

Taking into account the definitions (51) of H and $U^{\left(S\right)}=C^{\left(S\right)}\times_{3}S\in C^{M_{S}\times P\times N}$, given in Table 13 allows us to rewrite (50) as the contraction of the tensors H and $U^{\left(S\right)}$ along their common mode $m_{S}$:

(53) $$X^{\left(D\right)}=H\times_{3}^{1}U^{\left(S\right)}.$$

This contraction is another way to interpret the Tucker train model represented by means of Figure 6. From Equations (46) and (47), we deduce:

(54) $$\begin{array}{cc} x_{m_{R},p,n}^{\left(R\right)}= & \;\sum_{m_{S}}\sum_{r}h_{m_{R},m_{S}}^{\left(SR\right)}c_{m_{S},p,r}^{\left(S\right)}s_{n,r} \end{array}$$

(55) $$\begin{array}{cc} x_{m_{D},j,p,n}^{\left(D\right)}= & \;\sum_{m_{T}}\sum_{m_{R}}h_{m_{D},m_{T}}^{\left(RD\right)}c_{m_{T},j,m_{R}}^{\left(R\right)}x_{m_{R},p,n}^{\left(R\right)}. \end{array}$$

**Figure 7:**
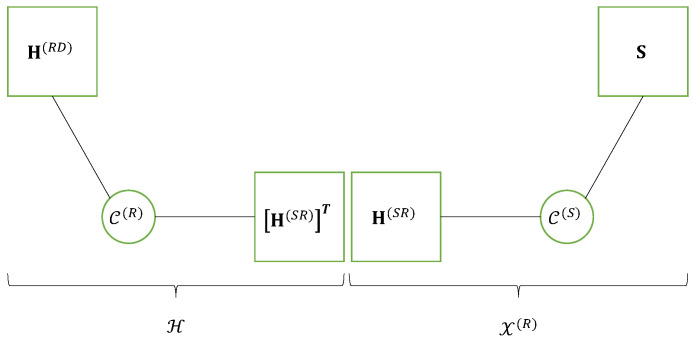
NTD model of a relaying system using TST codings.

Replacing $x_{m_{R},p,n}^{\left(R\right)}$ by its expressions (54) into (55) gives the signal received at destination by the $m_{D}$-th antenna, during the *n*-th symbol period of the *p*-th time-block (of the source) and *j*-th time block (of the relay):

(56) $$x_{m_{D},j,n,p}^{\left(D\right)}=\sum_{m_{T}}\sum_{m_{R}}\sum_{m_{S}}\sum_{r}h_{m_{D},m_{T}}^{\left(RD\right)}c_{m_{T},j,m_{R}}^{\left(R\right)}h_{m_{R},m_{S}}^{\left(SR\right)}c_{m_{S},p,r}^{\left(S\right)}s_{n,r},$$

where the blue color is associated with the sum over indices $m_{S}$ and *r* corresponding to the Tucker-(2,3) model of the tensor $X^{\left(R\right)}$, while the red color is used for the sum over indices $m_{T}$ and $m_{R}$ associated with the Tucker-(2,3) model of the effective channel H. The matrix factor $H^{\left(SR\right)}$ shared by both Tucker models is in green. Thus, the Tucker train model of the fourth-order tensor $X^{\left(D\right)}$, represented in Figure 6, can also be viewed as a nested Tucker decomposition (nTD) model, as illustrated by means of Figure 7. This figure is to be compared with Figure 4 of the nCPD model of the relaying system using SKRST coding.

**Remark** **6.** *Recalling that the third-order tensor $U^{\left(S\right)}\in C^{M_{S}\times P\times N}$ satisfies the Tucker-(1,3) model $\left[[C^{\left(S\right)};I_{M_{S}},I_{P},S]\right]$, we have:*

(57) $$u_{m_{S},p,n}^{\left(S\right)}=\;\sum_{r}c_{m_{S},p,r}^{\left(S\right)}s_{n,r}.$$

*From Equations (52) and (57), we can also write (56) as:*

(58) $$x_{m_{D},j,p,n}^{\left(D\right)}=\sum_{m_{S}}h_{m_{D},j,m_{S}}u_{m_{S},p,n}^{\left(S\right)},$$

*which is the scalar writing of the contraction operation (53).*

In conclusion, the signals received at destination form a fourth-order tensor $X^{\left(D\right)}\in C^{M_{D}\times J\times P\times N}$, which satisfies a Tucker train model, represented by means of Equation (50) and Figure 6. This model can also be viewed as a nTD model represented in Figure 7 corresponding to the nesting of two Tucker-(2,3) models. An extension of the multi-hop case can be found in [29].

**Remark** **7.** *If the DF protocol is employed at the relay, the tensors of signals received at the relay and destination nodes satisfy the following two Tucker-(2,3) models:*

(59) $$\begin{array}{cc} X^{\left(R\right)}= & \;C^{\left(S\right)}\times_{1}H^{\left(SR\right)}\times_{3}S\in C^{M_{R}\times P\times N} \end{array}$$

(60) $$\begin{array}{cc} X^{\left(D\right)}= & \;C^{\left(R\right)}\times_{1}H^{\left(RD\right)}\times_{3}\hat{S}\in C^{M_{D}\times J\times N}, \end{array}$$

*where $\hat{S}$ denotes the symbol matrix estimated at the relay before its encoding with the TST code $C^{\left(R\right)}\in C^{M_{T}\times J\times R}$. From the Tucker models (59) and (60), we deduce the following matrix unfoldings of $X^{\left(R\right)}$ and $X^{\left(D\right)}$:*

(61) $$\begin{array}{cc} X_{M_{R}N\times P}^{\left(R\right)}= & \;\left(H^{\left(SR\right)}⊗S\right)C_{M_{S}R\times P}^{\left(S\right)} \end{array}$$

(62) $$\begin{array}{cc} X_{M_{D}N\times J}^{\left(D\right)}= & \;\left(H^{\left(RD\right)}⊗\hat{S}\right)C_{M_{T}R\times J}^{\left(R\right)}. \end{array}$$

*Assuming the coding tensors $C^{\left(S\right)}$ and $C^{\left(R\right)}$ known at the relay and the destination, respectively, and choosing these tensors such as their unfoldings $C_{M_{S}R\times P}^{\left(S\right)}$ and $C_{M_{T}R\times J}^{\left(R\right)}$ are row-orthonormal, which implies the necessary conditions $P\geq M_{S}R$ and $J\geq M_{T}R$, the LS estimates of the Kronecker products between the channels and symbol matrices are calculated as:*

(63) $$\begin{array}{cc} \hat{H^{\left(SR\right)}⊗S}= & \;X_{M_{R}N\times P}^{\left(R\right)}{\left(C_{M_{S}R\times P}^{\left(S\right)}\right)}^{H} \end{array}$$

(64) $$\begin{array}{cc} \hat{H^{\left(RD\right)}⊗\hat{S}}= & \;X_{M_{D}N\times J}^{\left(D\right)}{\left(C_{M_{T}R\times J}^{\left(R\right)}\right)}^{H}. \end{array}$$

*These estimated Kronecker products can be used to estimate the channels and symbol matrices by means of the KronF algorithm; this will be detailed in a companion paper [12].*

### 5.4. Multi-User Relay System Using TST Codings

In this section, we propose an extension of the previous system to the multi-user case. Let us consider *Q* users equipped with $M_{q}$ antennas each, and assume that the *q*-th user, for q∈〈Q〉, sends the symbol matrix $S^{\left(q\right)}\in C^{N\times R_{q}}$ to the destination node equipped with $M_{D}$ antennas via the relay having $M_{R}$ receive and $M_{T}$ transmit antennas. Each user *q* codes his symbols to transmit using the TST code $C^{\left(q\right)}\in C^{M_{q}\times P\times R_{q}}$ to deliver the coded signals tensor $U^{\left(q\right)}=C^{\left(q\right)}\times_{3}S^{\left(q\right)}\in C^{M_{q}\times P\times N_{q}}$. The signals received from the *Q* users at the relay are concatenated in the tensor $X^{\left(R\right)}\in C^{M_{R}\times P\times N}$ such as:

(65) $$X^{\left(R\right)}={⊔}_{3}\left(U^{\left(q\right)}\times_{1}H^{\left(qR\right)}\right)={⊔}_{3}\left(C^{\left(q\right)}\times_{1}H^{\left(qR\right)}\times_{3}S^{\left(q\right)}\right),$$

where ${⊔}_{3}$ denotes the concatenation along the mode-3 of the *Q* tensors $U^{\left(q\right)}\times_{1}H^{\left(qR\right)}\in C^{M_{R}\times P\times N_{q}}$, for q∈〈Q〉, $N=\sum_{q=1}^{Q}N_{q}$, and $H^{\left(qR\right)}\in C^{M_{R}\times M_{q}}$ is the channel between user *q* and the relay. As in Table 13, the signals received at relay are coded by means of the TST code $C^{\left(R\right)}\in C^{M_{T}\times J\times M_{R}}$ and sent to destination via the channel $H^{\left(RD\right)}\in C^{M_{D}\times M_{T}}$, which gives:

(66) $$X^{\left(D\right)}=C^{\left(R\right)}\times_{1}H^{\left(RD\right)}\times_{3}^{1}X^{\left(R\right)}\in C^{M_{D}\times J\times P\times N}.$$

Compared with the previous system, the main difference is in the tensor $X^{\left(R\right)}$. Let us define the global source coding tensor $C^{\left(S\right)}\in C^{M_{S}\times P\times R}$, whose *p*-th lateral slice is given by $\mathrm{diag}\left(C_{.p.}^{\left(1\right)},\cdots ,C_{.p.}^{\left(Q\right)}\right)$, where $C_{.p.}^{\left(q\right)}\in C^{M_{q}\times R_{q}}$, with $M_{S}=\sum_{q}M_{q}$ and $R=\sum_{q}R_{q}$. We now define the global symbol and channel matrices containing the *Q* symbol and channel matrices as:

(67) $$S≜\left[S^{\left(1\right)},\cdots ,S^{\left(Q\right)}\right]\in C^{N\times R}\;,\;H^{\left(SR\right)}≜\left[H^{\left(1R\right)},\cdots ,H^{\left(QR\right)}\right]\in C^{M_{R}\times M_{S}}.$$

Then, $X^{\left(R\right)}$ defined in (65), can be rewritten as:

(68) $$X^{\left(R\right)}=C^{\left(S\right)}\times_{1}H^{\left(SR\right)}\times_{3}S.$$

**Figure 8:**
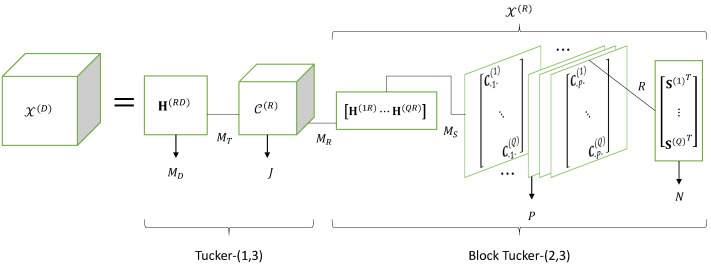
Block Tucker train of a multi-user relay system using TST codings.

From the above construction of the global coding tensor $C^{\left(S\right)}$ and symbol and channel matrices $\left(S,H^{\left(SR\right)}\right)$, we obtain an expression of $X^{\left(R\right)}$ similar to Equation (47). Equation (66) can be interpreted as the contraction between the tensor $C^{\left(R\right)}\times_{1}H^{\left(RD\right)}$ which satisfies the Tucker-(1,3) model $\left[[C^{\left(R\right)};H^{\left(RD\right)},I_{J},I_{M_{R}}]\right]$ with the tensor $X^{\left(R\right)}$, defined by means of Equation (68), which satisfies the block Tucker-(2,3) model $\left[[C^{\left(S\right)};H^{\left(SR\right)},I_{P},S]\right]$ along their common mode $m_{R}$. That corresponds to a new block Tucker train model illustrated in Figure 8, composed of the cascade of a Tucker-(1,3) model with a block Tucker-(2,3) model.

### 5.5. Parallel Multi-Relay Two-Hop Systems Using TST Codings

We now consider a two-hop system using *B* relays in parallel, operating sequentially, as illustrated in Figure 3d, with third- and fourth-order TST codings, $C^{\left(S\right)}\in C^{M_{S}\times P\times R}$ and $C^{\left(R\right)}\in C^{M_{T}\times J\times M_{R}\times B}$, at the source and relay nodes, respectively. Note that the coding tensor at each relay b∈〈B〉 is different. That explains dimension *B* of the tensor $C^{\left(R\right)}$, which contains along its fourth mode the relay numbers. Similarly, the source–relay and relay–destination channels depend on the relay, which explains the third-order channel tensors $H^{\left(SR\right)}\in C^{M_{R}\times M_{S}\times B}$ and $H^{\left(RD\right)}\in C^{M_{D}\times M_{T}\times B}$, respectively. The equations of this system are summarized in Table 14. The transmission is composed of B+1 steps, the first one corresponding to the transmission from the source to the relays; the *B* other steps correspond to a sequential transmission from the *B* relays to the destination.

**Table 14:** Two-hop systems using *B* relays in parallel with TST codings.

Ref. Signals	Symbols/Codings	Channels	Encoded/Received Signals	Dimensions
[34]	$S\in C^{N\times R}$			
		**First hop**		
Signals coded at source	$C^{\left(S\right)}\in C^{M_{S}\times P\times R}$		$U^{\left(S\right)}=C^{\left(S\right)}\times_{3}S$	$M_{S}\times P\times N$
Signals received at relay		$H^{\left(SR\right)}\in C^{M_{R}\times M_{S}\times B}$	$X^{\left(R\right)}=U^{\left(S\right)}\times_{1}^{2}H^{\left(SR\right)}$	$M_{R}\times P\times N\times B$
		**Second hop**		
Signals coded at relay	$C^{\left(R\right)}\in C^{M_{T}\times J\times M_{R}\times B}$		$U^{\left(R\right)}=C^{\left(R\right)}\times_{3}^{1}X^{\left(R\right)}$	$M_{T}\times J\times P\times N\times B$
Signals received at destination		$H^{\left(RD\right)}\in C^{M_{D}\times M_{T}\times B}$	$X^{\left(D\right)}=U^{\left(R\right)}\times_{1}^{2}H^{\left(RD\right)}$	$M_{D}\times J\times P\times N\times B$

Noting that $U^{\left(R\right)}\times_{1}^{2}H^{\left(RD\right)}=H^{\left(RD\right)}\times_{2}^{1}U^{\left(R\right)}$ and $U^{\left(S\right)}\times_{1}^{2}H^{\left(SR\right)}=H^{\left(SR\right)}\times_{2}^{1}U^{\left(S\right)}$, equations in Table 14 lead to the following fifth-order tensor $X^{\left(D\right)}\in C^{M_{D}\times J\times P\times N\times B}$ containing the signals received at destination:

(69) $$\begin{array}{cc} X^{\left(D\right)}= & H^{\left(RD\right)}\times_{2}^{1}U^{\left(R\right)}=H^{\left(RD\right)}\times_{2}^{1}\left(C^{\left(R\right)}\times_{3}^{1}X^{\left(R\right)}\right)\\ = & H^{\left(RD\right)}\times_{2}^{1}C^{\left(R\right)}\times_{3}^{1}\left(H^{\left(SR\right)}\times_{2}^{1}U^{\left(S\right)}\right)\\ = & H^{\left(RD\right)}\times_{2}^{1}C^{\left(R\right)}\times_{3}^{1}H^{\left(SR\right)}\times_{2}^{1}C^{\left(S\right)}\times_{3}^{2}S. \end{array}$$

This equation highlights the contraction operations represented by the mode-(i,j) products, denoted $\times_{2}^{1}$, $\times_{3}^{1}$ and $\times_{3}^{2}$. Each mode-(i,j) product is associated with a sum over the index shared by the tensors involved in the product. Equation (69) therefore implies sums over the indices $m_{T},m_{R},m_{S}$ and *r*. These sums lead to the following scalar expression of the signal $x_{m_{D},j,p,n,b}^{\left(D\right)}$ received at destination from relay *b* at the $m_{D}$-th antenna of the destination node during the *n*-th symbol period associated with the *p*-th code of the source and *j*-th code of the *b*-th relay:

(70) $$x_{m_{D},j,p,n,b}^{\left(D\right)}=\sum_{m_{T}}\sum_{m_{R}}\sum_{m_{S}}\sum_{r}h_{m_{D},m_{T},b}^{\left(RD\right)}\;c_{m_{T},j,m_{R},b}^{\left(R\right)}\;h_{m_{R},m_{S},b}^{\left(SR\right)}\;c_{m_{S},p,r}^{\left(S\right)}s_{n,r}.$$

Defining the effective channel tensor as:

(71) $$H=H^{\left(RD\right)}\times_{2}^{1}C^{\left(R\right)}\times_{3}^{1}H^{\left(SR\right)}\in C^{M_{D}\times J\times M_{S}\times B},$$

Equation (69) can also be written as:

(72) $$X^{\left(D\right)}=H\times_{3}^{1}U^{\left(S\right)}=H\times_{3}^{1}\left(C^{\left(S\right)}\times_{3}S\right).$$

**Figure 9:**
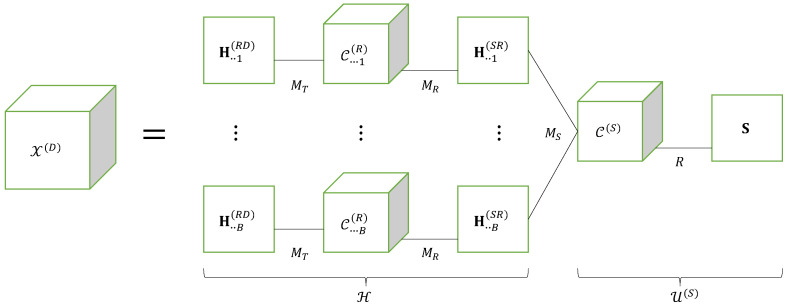
Coupled Tucker train model of a multi-relay system using TST codings.

This equation corresponds to *B* coupled Tucker trains, called a coupled Tucker train model, as illustrated by means of Figure 9. The coupling is due to the tensor $U^{\left(S\right)}$ of the signals coded at source, which is common in the tensors of signals transmitted by the *B* relays via the effective channel tensor H.

Comparing Figure 6 and Figure 9, we conclude that, for the multi-relay system, the signal received at destination is composed of *B* signals sequentially received from the *B* relays, which explains the *B* parallel branches in Figure 9. The multi-relay system allows one to increase the system diversity to estimate the information symbols due to the repetition of signals received at destination.

### 5.6. IRS-Assisted Two-Hop Systems Using SKRST Coding

In this section, we extend the relay-assisted two-hop system described in Section 5.1 to an IRS-assisted two-hop system, as represented in Figure 3g, with a single source.

In Table 15, we summarize the equations of an IRS-assisted uplink communication between a source and a BS, equipped with $M_{S}$ and $M_{D}$ antennas, respectively. The information symbols contained in the symbol matrix $S\in C^{N\times M_{S}}$ are coded by the source using SKRST coding and sent during *T* time slots, each time slot being composed of *N* symbol periods. The reflector cells are assumed to be varying at each time slot t∈〈T〉, and modeled by means of the matrix $G\in C^{T\times M_{I}}$, where $M_{I}$ is the number of cells.

**Table 15:** IRS-assisted two-hop system using SKRST coding.

Signals	Symbols/Coding	Channels	Received Signals	Dimensions
	$S\in C^{N\times M_{S}}$			
		**First hop**		
Signals coded at source	$C^{\left(S\right)}\in C^{P\times M_{S}}$		$U_{PN\times M_{S}}^{\left(S\right)}=C^{\left(S\right)}⋄S$	$PN\times M_{S}$
Signals received at IRS		$H^{\left(SI\right)}\in C^{M_{I}\times M_{S}}$	$X_{M_{I}\times PN}^{\left(I\right)}=H^{\left(SI\right)}U_{M_{S}\times PN}^{\left(S\right)}$	$M_{I}\times PN$
Signals reflected by IRS at time slot *t*		$G\in C^{T\times M_{I}}$	$U_{M_{I}\times PN}^{\left(I\right)}\left(t\right)=D_{t}\left(G\right)X_{M_{I}\times PN}^{\left(I\right)}$	$M_{I}\times PN$
			$=D_{t}\left(G\right)H^{\left(SI\right)}U_{M_{S}\times PN}^{\left(S\right)}$	
		**Second hop**		
Signals received at destination at time slot *t*		$H^{\left(ID\right)}\in C^{M_{D}\times M_{I}}$	$X^{\left(D\right)}\left(t\right)=H^{\left(ID\right)}U_{M_{I}\times PN}^{\left(I\right)}\left(t\right)$	$M_{D}\times PN$

The matrix equations in Table 15 are now reformulated using the tensor formalism. Similar to Equation (18), the signals received at IRS form a third-order tensor $X^{\left(I\right)}\in C^{M_{I}\times P\times N}$ such as:

(73) $$X^{\left(I\right)}=U^{\left(S\right)}\times_{1}H^{\left(SI\right)}\;\;⟺\;\;X^{\left(I\right)}=I_{M_{S}}\times_{1}H^{\left(SI\right)}\times_{2}C^{\left(S\right)}\times_{3}S,$$

with $U^{\left(S\right)}\in C^{M_{S}\times P\times N}$ defined in (16). From equations in Table 15, it is easy to derive the following equation satisfied by the signals received at destination at time slot *t*:

(74) $$\begin{array}{cc} X^{\left(D\right)}\left(t\right)= & \;H^{\left(ID\right)}D_{t}\left(G\right)H^{\left(SI\right)}U_{M_{S}\times PN}^{\left(S\right)}\\ = & \;H^{\left(ID\right)}D_{t}\left(G\right)H^{\left(SI\right)}{\left(C^{\left(S\right)}⋄S\right)}^{T}\in C^{M_{D}\times PN}. \end{array}$$

This equation can be interpreted as the *t*-th lateral slice of the contracted tensor $X_{c}^{\left(D\right)}\in C^{M_{D}\times T\times PN}$.

Let us define the third-order effective channel tensor $H\in C^{M_{D}\times T\times M_{S}}$, whose *t*-th lateral slice is given by:

(75) $$H_{.t.}=H^{\left(ID\right)}D_{t}\left(G\right)H^{\left(SI\right)}\in C^{M_{D}\times M_{S}}.$$

This tensor H satisfies the CPD model $\left[[H^{\left(ID\right)},G,{\left(H^{\left(SI\right)}\right)}^{T};M_{I}]\right]$. Equation (74) can then be interpreted as the contraction between tensors H and $U^{\left(S\right)}$ along their common mode $m_{S}$; that means:

(76) $$X^{\left(D\right)}=H\times_{3}^{1}U^{\left(S\right)}.$$

**Figure 10:**
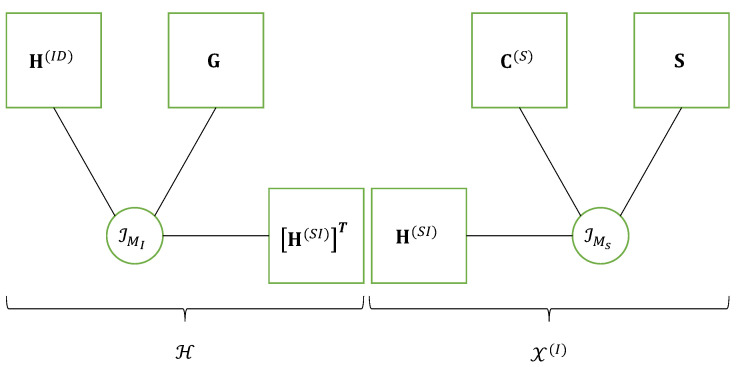
Nested CPD model of an IRS-assisted system using SKRST coding.

This equation is to be compared with (40) and (72). Since H and $X^{\left(I\right)}$ satisfy two third-order CPD models sharing the matrix factor $H^{\left(SI\right)}$, Equation (74) can also be interpreted as a nested CPD model, as illustrated by means of Figure 10.

This figure is similar to Figure 4, highlighting the following correspondences:

(77) $$\begin{array}{cc} (H^{\left(RD\right)},C^{\left(R\right)},H^{\left(SR\right)},C^{\left(S\right)},S;X^{\left(R\right)})\;\;⟷ & \;\;(H^{\left(ID\right)},G,H^{\left(SI\right)},C^{\left(S\right)},S;X^{\left(I\right)}) \end{array}$$

(78) $$\begin{array}{cc} (M_{R},M_{S},P,J)\;\;⟷ & \;\;(M_{I},M_{S},P,K). \end{array}$$

We can conclude that the coding matrix $C^{\left(R\right)}\in C^{J\times M_{R}}$ at the relay in Figure 4 is replaced by the reflector matrix $G\in C^{K\times M_{I}}$ in Figure 10, with the code length *J* replaced by the number *T* of time slots, meaning that the code diversity is replaced by the time diversity represented by the dimensions *J* and *T*, respectively, in the tensor $X^{\left(D\right)}$ of signals received at destination.

Assuming the coding matrix $C^{\left(S\right)}$ and the reflector matrix G are known at destination, the nested CPD model of the received signal’s tensor, $X^{\left(D\right)}$, can be exploited to develop semi-blind receivers for jointly estimating the individual channels and symbol matrices $\left(H^{\left(SI\right)},H^{\left(ID\right)},S\right)$. Such receivers will be presented in a companion paper [12].

## 6. Conclusions and Perspectives

In this paper, we first introduced basic tensor operations commonly used in the exploitation of tensor models. The Tucker decomposition and CPD, which are the basis of several of the models highlighted in this paper for the design of different cooperative communication systems, were recalled. Then, we described the main characteristics allowing us to classify cooperative wireless communication systems, before illustrating several architectures of relay-, IRS- and UAV-assisted communication networks. An overview of several cooperative systems has been provided in a synthetic and comparative way, highlighting the characteristics of each system in terms of modulation, technology and coding employed, and tensor models for representing the received signals. Then, we provided an overview of the main codings proposed in the context of both point-to-point and multi-hop systems. Finally, to illustrate the tensor-based approach for the design of cooperative systems, several two-hop systems have been described in a didactic and unified way, using different codings and by detailing, for each system, the signals coded, transmitted and received both at the relay or IRS and at the destination. Some of the presented systems are extensions of existing ones, which led to the introduction of several new tensor models.

In a companion paper under preparation, a focus will be made on how these tensor models can be exploited to develop semi-blind receivers for jointly estimating the transmitted information symbols, the individual channels and eventually the channels parameters, like DoA and DoD angles. The uniqueness of the tensor models of each system and parameter identifiability conditions for each estimation algorithm will be analyzed and compared. Monte Carlo simulation results will be provided to illustrate and compare the effectiveness of the considered cooperative systems and associated semi-blind receivers.

As perspectives of this work, we plan to pursue the tensor-based approach to develop new IRS- and UAV-assisted systems using different codings and under various configurations in terms of massive MIMO and DD-DP channels, with the objective of reducing the parametric complexity of tensor models and the computational complexity of receivers.

## Figures and Tables

**Table 1:** Notation.

Symbols	Definitions
K=R or C	set of real or complex numbers
〈N〉≜{1,⋯,N}	Set of first *N* integers
${\underline{i}}_{N}≜\left\{i_{1},\cdots ,i_{N}\right\}$	Set of *N* indices
${\underline{I}}_{N}≜I_{1}\times \cdots \times I_{N}$	Size of an *N*th-order tensor
*a*, a, A, A	Scalar, column vector, matrix, tensor
$a_{{\underline{i}}_{N}}=a_{i_{1},i_{2},\cdots ,i_{N}}$ or ${\left[A\right]}_{i_{1},i_{2},\cdots ,i_{N}}$	$\left(i_{1},i_{2},\cdots ,i_{N}\right)$-th element of $A\in K^{{\underline{I}}_{N}}$
$A^{T}$	Transpose of A
$A^{*}$	Complex conjugate of A
$A^{†}$	Moore-Penrose pseudo-inverse of A
$A_{i\cdot }$ $\left(A_{\cdot j}\right)$	*i*-th (*j*-th) row (column) of $A\in K^{I\times J}$
$A_{\cdots i_{n}\cdots }$ or $A_{\left(i_{n}\right)}$	Mode-*n* tensor slice
$A_{I_{1}\cdots I_{N-1}\times I_{N}}$	Tall mode-*N* matrix unfolding of A
$e_{n}^{\left(N\right)}$	*n*th canonical basis vector of the Euclidean space $R^{N}$
vec(·)	Vectorization operator
diag(·)	Diagonalization operator which forms a matrix from its vector argument
$D_{i}\left(A\right)=diag\left(A_{i.}\right)$	Diagonal matrix whose diagonal entries are the elements of the *i*-th row of $A\in K^{I\times J}$
bdiag(·)	Block-diagonalization operator
$\langle \cdot ,\cdot \rangle$	Inner product
${∥\cdot ∥}_{F}$	Frobenius norm
∘	Outer product
⋄	Khatri-Rao product
⊗	Kronecker product
⋈	Block Kronecker product
$\times_{n}$	Mode-*n* product
$\times_{m}^{n}$	Contraction operation
${⊔}_{n}$	Concatenation operation along mode *n*

**Table 2:** Two multiplications with tensors.

Tensors	Operations	Definitions
$X\in K^{{\underline{I}}_{P}},A\in K^{J\times I_{p}}$	$Y=X\times_{p}A$	$y_{i_{1},\cdots ,i_{p-1}\;,j,\;i_{p+1},\cdots ,i_{P}}=\sum_{i_{p}}a_{j,i_{p}}x_{{\underline{i}}_{P}}$
$X\in K^{{\underline{I}}_{P}},Y\in K^{{\underline{J}}_{N}}$	$Z=X\times_{p}^{n}Y$	$z_{i_{1},\cdots ,i_{p-1},\;i_{p+1},\cdots ,i_{P},j_{1},\cdots ,j_{n-1},\;j_{n+1},\cdots ,j_{N}}=$
with $I_{p}=J_{n}=K$		$\sum_{k=1}^{K}\;a_{i_{1},\cdots ,i_{p-1},\;k,\;i_{p+1},\cdots ,i_{P}}\;b_{j_{1},\cdots ,j_{n-1},\;k,\;j_{n+1},\cdots ,j_{N}}$

**Table 3:** Matrix unfoldings of products with tensors.

Dimensions	Products	Matrix Unfoldings	Dimensions of Resulting Tensors
$X\in K^{I\times J\times K},A\in K^{L\times K}$	$Y=X\times_{3}A$	$Y_{IJ\times L}=X_{IJ\times K}A^{T}$	$Y\in K^{I\times J\times L}$
$X\in K^{I\times J\times K\times M},A\in K^{L\times K}$	$Y=X\times_{3}A$	$Y_{IJM\times L}=X_{IJM\times K}A^{T}$	$Y\in K^{I\times J\times L\times M}$
$X\in K^{I\times J\times P},Y\in K^{L\times M\times N}$ with P=M=K	$Z=X\times_{p}^{m}Y$	$z_{i,j,l,n}=\sum_{k=1}^{K}\;a_{i,j,k}\;b_{l,k,n}$ $Z_{IJ\times LN}=X_{IJ\times K}Y_{K\times LN}$	$Z\in K^{I\times J\times L\times N}$

**Table 4:** Outer products of vectors, matrices and tensors.

Vectors/Matrices/Tensors	Outer Products	Spaces	Orders
$u^{\left(p\right)}\in K^{I_{p}}$, p∈〈P〉	$\overset{P}{\underset{p=1}{\circ }}u^{\left(p\right)}$	$K^{{\underline{I}}_{P}}$	*P*
$A^{\left(p\right)}\in K^{I_{p}\times J_{p}},p\in \langle P\rangle$	$\overset{P}{\underset{p=1}{\circ }}A^{\left(p\right)}$	$K^{I_{1}\times J_{1}\times \cdots \times I_{P}\times J_{P}}$	2P
$A\in K^{{\underline{I}}_{P}},B\in K^{{\underline{J}}_{N}}$	A∘B	$K^{{\underline{I}}_{P}\times {\underline{J}}_{N}}$	P+N
$A^{\left(p\right)}\in K^{{\underline{J}}_{N_{p}}},p\in \langle P\rangle$	$\overset{P}{\underset{p=1}{\circ }}A^{\left(p\right)}$	$K^{{\underline{J}}_{N_{1}}\times \cdots \times {\underline{J}}_{N_{P}}}$	$\sum_{p=1}^{P}N_{p}$

**Table 5:** Scalar elements of outer products.

Matrices/Tensors	Outer Products	Elements of X	Indices
$A^{\left(p\right)}\in K^{I_{p}\times J_{p}}$	$X=\overset{P}{\underset{p=1}{\circ }}A^{\left(p\right)}$	$x_{i_{1}j_{1}\cdots i_{P}j_{P}}=\prod_{p=1}^{P}a_{i_{p}j_{p}}^{\left(p\right)}$	$i_{p}\in \langle I_{p}\rangle \;\;,\;\;p\in \langle P\rangle$
			$j_{p}\in \langle J_{p}\rangle \;\;,\;\;p\in \langle P\rangle$
$A\in K^{{\underline{I}}_{P}},B\in K^{{\underline{J}}_{N}}$	X=A∘B	$x_{{\underline{i}}_{P}\;,\;{\underline{j}}_{N}}=a_{{\underline{i}}_{P}}b_{{\underline{j}}_{N}}$	$i_{p}\in \langle I_{p}\rangle \;\;,\;\;p\in \langle P\rangle$
			$j_{n}\in \langle J_{n}\rangle \;\;,\;\;n\in \langle N\rangle$
$A^{\left(p\right)}\in K^{{\underline{I}}_{N_{p}}}$	$X=\overset{P}{\underset{p=1}{\circ }}A^{\left(p\right)}$	$x_{{\underline{i}}_{N_{1}}\;,\;\cdots \;,\;{\underline{i}}_{N_{P}}}=\prod_{p=1}^{P}a_{{\underline{i}}_{N_{p}}}^{\left(p\right)}$	${\underline{i}}_{N_{p}}=\left(i_{1},\cdots ,i_{N_{p}}\right)$
			p∈〈P〉

**Table 6:** Tucker decomposition and CPD of an *N*th-order tensor.

Tucker Decomposition		CPD
	$X\in K^{{\underline{I}}_{N}}$	
$G\in K^{{\underline{R}}_{N}}$		
$A^{\left(n\right)}\in K^{I_{n}\times R_{n}}$		$A^{\left(n\right)}\in K^{I_{n}\times R}$
$x_{{\underline{i}}_{N}}=\sum_{r_{1}=1}^{R_{1}}\cdots \sum_{r_{N}=1}^{R_{N}}g_{r_{1},\cdots ,r_{N}}\prod_{n=1}^{N}a_{i_{n},r_{n}}^{\left(n\right)}$	Scalar writing	$x_{{\underline{i}}_{N}}=\sum_{r=1}^{R}\prod_{n=1}^{N}a_{i_{n},r}^{\left(n\right)}$
	Writing with	
$X=G\overset{N}{\underset{n=1}{\times }}A^{\left(n\right)}$	mode-*n* products	$X=I_{R}\overset{N}{\underset{n=1}{\times }}A^{\left(n\right)}$
$X=\sum_{r_{1}=1}^{R_{1}}\cdots \sum_{r_{N}=1}^{R_{N}}g_{r_{1},\cdots ,r_{N}}\overset{N}{\underset{n=1}{\circ }}A_{.r_{n}}^{\left(n\right)}$	Outer products	$X=\sum_{r=1}^{R}\overset{N}{\underset{n=1}{\circ }}A_{.r}^{\left(n\right)}$
	Matricization	
$\left(\underset{n\in S_{1}}{⊗}A^{\left(n\right)}\right)G_{S_{1};S_{2}}{\left(\underset{n\in S_{2}}{⊗}A^{\left(n\right)}\right)}^{T}$	$X_{S_{1};S_{2}}=$	$\left(\underset{n\in S_{1}}{⋄}A^{\left(n\right)}\right){\left(\underset{n\in S_{2}}{⋄}A^{\left(n\right)}\right)}^{T}$

**Table 7:** Tucker decomposition and CPD of a third-order tensor.

Tucker Decomposition		CPD
	$X\in K^{I\times J\times K}$	
$G\in K^{P\times Q\times S},A\in K^{I\times P},B\in K^{J\times Q},C\in K^{K\times S}$		$A\in K^{I\times R},B\in K^{J\times R},C\in K^{K\times R}$
$x_{i,j,k}=\sum_{p=1}^{P}\sum_{q=1}^{Q}\sum_{s=1}^{S}g_{pqs}a_{ip}b_{jq}c_{ks}$	Scalar writing	$x_{i,j,k}=\sum_{r=1}^{R}a_{ir}b_{jr}c_{kr}$
	Writing with	
$X=G\times_{1}A\times_{2}B\times_{3}C$	mode-*n* products	$X=I_{R}\times_{1}A\times_{2}B\times_{3}C$
$X=\sum_{p=1}^{P}\sum_{q=1}^{Q}\sum_{s=1}^{S}g_{pqs}A_{.p}\circ B_{.q}\circ C_{.s}$	Outer products	$X=\sum_{r=1}^{R}A_{.r}\circ B_{.r}\circ C_{.r}$
$X_{IJ\times K}=\left(A⊗B\right)G_{PQ\times S}C^{T}$		$X_{IJ\times K}=\left(A⋄B\right)C^{T}$
$X_{JK\times I}=\left(B⊗C\right)G_{QS\times P}A^{T}$	Matrix unfoldings	$X_{JK\times I}=\left(B⋄C\right)A^{T}$
$X_{KI\times J}=\left(C⊗A\right)G_{SP\times Q}B^{T}$		$X_{KI\times J}=\left(C⋄A\right)B^{T}$

**Table 8:** Tucker-(2,3) and Tucker-(1,3) models.

Tucker-(2,3) Model		Tucker-(1,3) Model
	$X\in K^{I\times J\times K}$	
$G\in K^{P\times Q\times K}$		$G\in K^{P\times J\times K}$
$A\in K^{I\times P},B\in K^{J\times Q},C=I_{K}$		$A\in K^{I\times P},B=I_{J},C=I_{K}$
$x_{ijk}=\sum_{p=1}^{P}\sum_{q=1}^{Q}g_{pqk}a_{ip}b_{jq}$	Scalar expression	$x_{ijk}=\sum_{p=1}^{P}g_{pjk}a_{ip}$
$X=G\times_{1}A\times_{2}B$	With mode-*n* products	$X=G\times_{1}A$
$X_{IJ\times K}=\left(A⊗B\right)G_{PQ\times K}$		$X_{IJ\times K}=\left(A⊗I_{J}\right)G_{PJ\times K}$
$X_{JK\times I}=\left(B⊗I_{K}\right)G_{QK\times P}A^{T}$	Matrix unfoldings	$X_{JK\times I}=G_{JK\times P}A^{T}$
$X_{KI\times J}=\left(I_{K}⊗A\right)G_{KP\times Q}B^{T}$		$X_{KI\times J}=\left(I_{K}⊗A\right)G_{KP\times J}$

**Table 10:** Codings.

Codings	Symbol Matrices	Coding Matrices/Tensors	Encoded Signals
KRST	$S\in C^{M\times R}$	$C\in C^{M\times M}\;\;,\;\;W\in C^{P\times M}$	$V=S^{T}\;C\in C^{R\times M}\;\;,\;\;v_{r,m}=\sum_{l=1}^{M}s_{l,r}\;c_{l,m}$
[50]			$U=V\underset{m}{⊙}W=S^{T}\;C\;\underset{m}{⊙}\;W\in C^{R\times M\times P}$
			$u_{r,m,p}=v_{r,m}w_{p,m}=\sum_{l=1}^{M}s_{l,r}\;c_{l,m}w_{p,m}$
Simplified KRST	$S\in C^{N\times M}$	$C\in C^{P\times M}$	$U=C⋄S\in C^{PN\times M}\;⟺\;U\in C^{M\times P\times N}$
[21]			$u_{m,p,n}=c_{p,m}s_{n,m}$
DKRSTF	$S\in C^{N\times M}$	$A\in C^{F\times M}\;\;,\;\;C\in C^{M\times M}$	$U=V_{FN\times M}\underset{m}{⊙}W$
		$W\in C^{P\times M}\;\;,\;\;V\in C^{F\times N\times M}$	$=\left(A⋄S\right)C\;\underset{m}{⊙}\;W\in C^{F\times N\times M\times P}$
[51]		$v_{f,n,m}=\sum_{l=1}^{M}a_{f,l}s_{n,l}c_{l,m}$	$u_{f,n,m,p}=\sum_{l=1}^{M}a_{f,l}s_{n,l}c_{l,m}w_{p,m}$
TST	$S\in C^{N\times R}$	$C\in C^{M\times R\times J}$	$U=C\times_{2}S\in C^{M\times N\times J}$
[52]			$u_{m,n,j}=\sum_{r=1}^{R}c_{m,r,j}\;s_{n,r}$
TSTF	$S\in C^{N\times R}$	$C\in C^{M\times R\times F\times P\times J}$	$U=C\times_{2}S\in C^{M\times N\times F\times P\times J}$
[15]			$u_{m,n,f,p,j}=\sum_{r=1}^{R}c_{m,r,f,p,j}s_{n,r}$
Simplified TSTF	$S\in C^{N\times R\times F}$	$C\in C^{M\times R\times F\times P}$	$U=C\times_{2}S\in C^{M\times N\times F\times P}$
[47]			$u_{m,n,f,p}=\sum_{r=1}^{R}c_{m,r,f,p}s_{n,r,f}$
MSMKron	$S=\overset{Q}{\underset{q=1}{⊗}}S^{\left(q\right)}\in C^{N\times R}$		$s_{n,r}=\prod_{q=1}^{Q}s_{n_{q},r_{q}}^{\left(q\right)}\;,\;\;\;r\in \langle R\rangle \;,\;\;\;n\in \langle N\rangle$
[25]	$S^{\left(q\right)}\in C^{N_{q}\times R_{q}}$		$r=r_{Q}^{\left(Q\right)}+\left(r_{Q-1}^{\left(Q-1\right)}-1\right)R_{Q}+\cdots +\left(r_{1}^{\left(1\right)}-1\right)\prod_{q=2}^{Q}R_{q}$
	$R=\prod_{q=1}^{Q}R_{q}\;,\;N=\prod_{n=1}^{Q}n_{q}$		$n=n_{Q}^{\left(Q\right)}+\left(n_{Q-1}^{\left(Q-1\right)}-1\right)N_{Q}+\cdots +\left(n_{1}^{\left(1\right)}-1\right)\prod_{q=2}^{Q}n_{q}$
MSMKR	$S=\overset{Q}{\underset{q=1}{⋄}}S^{\left(q\right)}\in C^{N\times R}$		$s_{n,r}=\prod_{q=1}^{Q}s_{n_{q},r}^{\left(q\right)}\;,\;\;\;r\in \langle R\rangle \;,\;\;\;n\in \langle N\rangle$
[25]	$S^{\left(q\right)}\in C^{N_{q}\times R}$		$n=n_{Q}^{\left(Q\right)}+\left(n_{Q-1}^{\left(Q-1\right)}-1\right)N_{Q}+\cdots +\left(n_{1}^{\left(1\right)}-1\right)\prod_{q=2}^{Q}N_{q}$
	$N=\prod_{q=1}^{Q}N_{q}$		
SKRST-MSMKR	$S=\overset{Q}{\underset{q=1}{⋄}}S^{\left(q\right)}\in C^{N\times M}$	$C\in C^{P\times M}$	$U=C⋄S\in C^{PN\times M}$
[53]	$S^{\left(q\right)}\in C^{N_{q}\times M},N=\prod_{q=1}^{Q}N_{q}$		$u_{p,n_{1},\cdots ,n_{Q},m}=c_{p,m}\prod_{q=1}^{Q}s_{n_{q},m}^{\left(q\right)}$
TST-MSMKron	$S=\overset{Q}{\underset{q=1}{⊗}}S^{\left(q\right)}\in C^{N\times R}$	$C\in C^{M\times R_{1}\cdots \times R_{Q}\times P}$	$U=C\times_{1}I_{M}\times_{2}S^{\left(1\right)}\times_{3}\cdots \times_{Q+1}S^{\left(Q\right)}\times_{Q+2}I_{P}\in C^{M\times N_{1}\cdots \times N_{Q}\times P}$
[54]	$S^{\left(q\right)}\in C^{N_{q}\times R_{q}}$		$u_{m,n_{1},\cdots ,n_{Q},p}=\sum_{r_{1}=1}^{R_{1}}\cdots \sum_{r_{Q}=1}^{R_{Q}}c_{m,r_{1},\cdots ,r_{Q},p}\prod_{q=1}^{Q}s_{n_{q},r_{q}}^{\left(q\right)}$

## Data Availability

Not applicable.

## Abbreviations

The following abbreviations are used in this manuscript:

AF	Amplify-and-forward
CPD	Canonical polyadic decomposition
DF	Decode-and-forward
DoA	Direction of arrival
DoD	Direction of departure
IRS	Intelligent reflecting surface
KronF	Kronecker factorization
KRF	Khatri-Rao factorization
KRST	Khatri-Rao space-time
KRSTF	Khatri-Rao space-time-frequency
MIMO	Multiple-input multiple-output
MSMKR	Multiple symbol matrices Khatri-Rao product
MSMKron	Multiple symbol matrices Kronecker product
mmW	Millimeter-wave
OFDM	Orthogonal frequency-division multiplexing
PARAFAC	Parallel factor analysis
SKRST	Simplified Khatri-Rao space-time
STF	Space-time-frequency
SVD	Singular-value decomposition
TST	Tensor space-time
TSTF	Tensor space-time-frequency
UAV	Unmanned aerial vehicular
